# Particulate matter composition drives differential molecular and morphological responses in lung epithelial cells

**DOI:** 10.1093/pnasnexus/pgad415

**Published:** 2023-12-28

**Authors:** Sean M Engels, Pratik Kamat, G Stavros Pafilis, Yukang Li, Anshika Agrawal, Daniel J Haller, Jude M Phillip, Lydia M Contreras

**Affiliations:** McKetta Department of Chemical Engineering, University of Texas at Austin, Austin, TX 78712, USA; Department of Chemical and Biomolecular Engineering, Johns Hopkins University, Baltimore, MD 21218, USA; McKetta Department of Chemical Engineering, University of Texas at Austin, Austin, TX 78712, USA; Department of Biology, Johns Hopkins University, Baltimore, MD 21218, USA; Department of Chemical and Biomolecular Engineering, Johns Hopkins University, Baltimore, MD 21218, USA; Department of Chemical and Biomolecular Engineering, North Carolina State University, Raleigh, NC 27606, USA; Department of Chemical and Biomolecular Engineering, Johns Hopkins University, Baltimore, MD 21218, USA; Institute for Nanobiotechnology, Johns Hopkins University, Baltimore, MD 21218, USA; Department of Biomedical Engineering, Johns Hopkins University, Baltimore, MD 21218, USA; Department of Oncology, Sidney Kimmel Comprehensive Cancer Center, Baltimore, MD 21231, USA; McKetta Department of Chemical Engineering, University of Texas at Austin, Austin, TX 78712, USA; Institute for Cellular and Molecular Biology, The University of Texas at Austin, Austin, TX, 78712, USA

**Keywords:** systems biology, transcriptomics, cell morphology, cell phenotyping, air pollution

## Abstract

Particulate matter (PM) is a ubiquitous component of air pollution that is epidemiologically linked to human pulmonary diseases. PM chemical composition varies widely, and the development of high-throughput experimental techniques enables direct profiling of cellular effects using compositionally unique PM mixtures. Here, we show that in a human bronchial epithelial cell model, exposure to three chemically distinct PM mixtures drive unique cell viability patterns, transcriptional remodeling, and the emergence of distinct morphological subtypes. Specifically, PM mixtures modulate cell viability, DNA damage responses, and induce the remodeling of gene expression associated with cell morphology, extracellular matrix organization, and cellular motility. Profiling cellular responses showed that cell morphologies change in a PM composition-dependent manner. Finally, we observed that PM mixtures with higher cadmium content induced increased DNA damage and drove redistribution among morphological subtypes. Our results demonstrate that quantitative measurement of individual cellular morphologies provides a robust, high-throughput approach to gauge the effects of environmental stressors on biological systems and score cellular susceptibilities to pollution.

**Significance Statement**

Air pollution is linked to multiple life-threatening pathologies including lung cancer and atherosclerotic diseases. A core component of air pollution is particulate matter (PM). Previous studies have not fully addressed that specific PM compositions elicit unique cellular responses at single-cell level. Additionally, these studies have predominantly focused on molecular responses. Combining transcriptional changes, pathway remodeling, and changes in cellular morphologies, we show that cell morphology is an indicator of PM-induced cellular damage and provide a robust framework to profile single-cell responses and susceptibility to PM mixtures.

## Introduction

Ambient air pollution threatens human health through direct links to chronic illnesses and premature deaths. High pollution levels are associated with elevated incidences of ischemic heart disease, lung cancer, aggravated asthma, chronic obstructive pulmonary disease (COPD), stroke, and adverse birth outcomes (1–6). In 2019, it was estimated that 6.67 million deaths could be attributed to air pollution exposure worldwide (7). Particulate matter (PM), which consists of microscopic solids and liquid droplets, is an important component of ambient air pollution and is widely studied in the context of global health (8). These particulates and their precursor chemicals are emitted from many natural and man-made sources, including volcanic activity, burning of biomass, vehicle emissions, coal-burning powerplants, and other industrial activities (9).

Studies have identified strong associations between PM size and different biological responses (10, 11). However, a key challenge in elucidating the effects of PM exposure is that PM chemical composition can vary greatly across geographical areas and environments, as there are various anthropogenic and biogenic contributors that emit different chemical species (12–14). These inherent geographical differences of PM can impose challenges toward understanding the different influences at the cellular and molecular levels, since the biological effects can vary with chemical composition. Studies using lung cell models such as A549, BEAS-2B, or primary airway epithelial cells have demonstrated the impact that different PM mixtures and pollutants can have on cellular pathway remodeling (11, 15). For example, studies using a bronchial epithelial cell model, BEAS-2B, exposed to mixtures of either secondary organic aerosol or aerosolized formaldehyde showed unique molecular responses and pathway remodeling (16, 17). Additionally, other studies have investigated the induction of oxidative stress due to PM exposure and have highlighted unique regulatory pathways that contribute to the proinflammatory response (15, 18). However, advances in high-throughput sequencing and quantitative morphological phenotyping to investigate the effects of multiple PM mixtures on the cellular and molecular level could allow for prediction and faster analysis of the effects of pollution mixtures on human health.

Different exposure methods have also provided insights into the biological effects of PM, including liquid-submerged exposures (19), air–liquid interface exposures (ALI) (20, 21), and pseudo-air–liquid-interface exposures (19). It is worth noting that these vary in cost, physiological relevance, and throughput. Studies have also looked at a variety of environmental pollutants, including PM_10_, PM_2.5_, and PM_0.1_ (PM with aerodynamic diameters of <10, 2.5, and 0.1 µm, respectively) collected from cities including Beijing, Milan, Seoul, and others (22–27). Organic and aqueous extractions of PM have also been investigated along with individual components or pollution types including secondary organic aerosols, diesel exhaust particles, volcanic ash, and metals. However, the results of these studies vary greatly, in part, due to their use of different cell models, exposure times and protocols, and PM types that are often not fully characterized. All these factors introduce challenges to drawing meaningful comparisons of the biological effects of different PM types. Thus, improving our methods to simultaneously map cellular and molecular effects of different PM mixtures using new high-throughput technologies continues to be an important area of research.

Previous studies looking at air-pollution-induced pathway remodeling via transcriptomics have found changes in regulatory pathways that control cellular morphology, including significant alterations in cholesterol synthesis pathways of bronchial epithelial cells that result in distinct morphological changes (17, 28). By extension, these types of studies indicate that decreases in cell size could be used as a biomarker of toxicity (17). Overall, cellular and nuclear morphology is linked to upstream changes in gene expression and cellular dysfunction (29, 30), with significant pathway remodeling in cell death programs, apoptotic pathways, extracellular matrix (ECM) interactions, and cytoskeleton structures (30–32). In the context of aging, changes in cell and nuclear sizes, as well as irregularities in cell shapes associate strongly with fundamental defects and senescence (33, 34). While long-term pollutant exposures of lung cells are linked to increased senescence, it is unclear how short-term exposures modulate cellular responses based on molecular or morphological phenotypes (35).

Here, we expose the BEAS-2B human bronchial epithelial cell model to three well-characterized and compositionally unique PM mixtures available from the National Institute of Standards and Technology (NIST): urban (SRM1648a), fine (SRM 2786), and diesel exhaust (SRM 2975). These mixtures have differing levels of important components, such as lead, cadmium, and nitro-polycyclic aromatic hydrocarbons (nitro-PAHs; Table 1 and Dataset S1). Exposures were performed at multiple concentrations ranging from 31 to 1,000 µg/mL for 24 h to investigate the effects of multiple PM types on human lung-epithelial cells. We performed liquid-submerged exposures given the high-throughput nature of the method relative to ALI. Following exposures, we measured transcriptional changes to identify specific PM-composition-dependent remodeling of molecular pathways. In parallel, we performed morphological analysis of cells at baseline and after PM exposures to develop a robust single-cell platform to profile cellular responses and the emergence of functional subtypes of cells. Together our study provides a multiscale approach to quantify molecular and morphological responses to several relevant PM mixtures. Additionally, we show that we can quantify cell morphology to score cellular susceptibility to PM exposure, offering a new tool for understanding the cellular effects of environmental stressors. To validate this, we isolated single-cell clones to show that subclones exhibit differential morphological responses which associated with differences in susceptibility.

**Table 1.** Select compositional differences between PM types.

Component	Urban PM mass fraction (mg/kg)	Fine PM mass fraction (mg/kg)	Diesel exhaust PM mass fraction (mg/kg)
Cadmium	73.7	4.34	—
Lead	6,550	286	—
Nitro-PAHs	0.73962	0.99598	45.907

Data listed as reported by NIST in mg/kg of total PM mass. No cadmium or lead concentrations were reported for SRM 2975.

## Results

### Cellular viability is differentially modulated by unique PM mixtures

We measured the survival of BEAS-2B cells following exposure to three individual PM mixtures sourced from NIST (urban, fine, and diesel exhaust) to quantify changes in toxicity to cells. The urban and fine samples contain PM collected over extended periods from two different cities, St Louis, Missouri and Prague, Czech Republic, respectively. The diesel exhaust sample was collected from the exhaust of a diesel-powered engine. Importantly, these mixtures exhibit major differences in several components; for instance, the mass fractions of lead, cadmium, and nitro-PAHs vary by at least an order of magnitude between at least two of the samples (Table 1). A complete comparison of reported compositional data is given in Dataset S1. Interestingly, cadmium and lead are both highly toxic metals that can be found in air pollution from manufacturing of batteries, cigarette smoke, metal processing, and the production of plastics (9, 36), while nitro-PAHs are primarily emitted from combustion of diesel fuel and have been shown to have mutagenic and genotoxic properties (37). Diesel exhaust is a major component of air pollution in urban areas resulting from heavy traffic, and diesel engines emit more particles and 10-times higher levels of nitro-PAHs than gasoline engines (38).

**Fig. 1.**
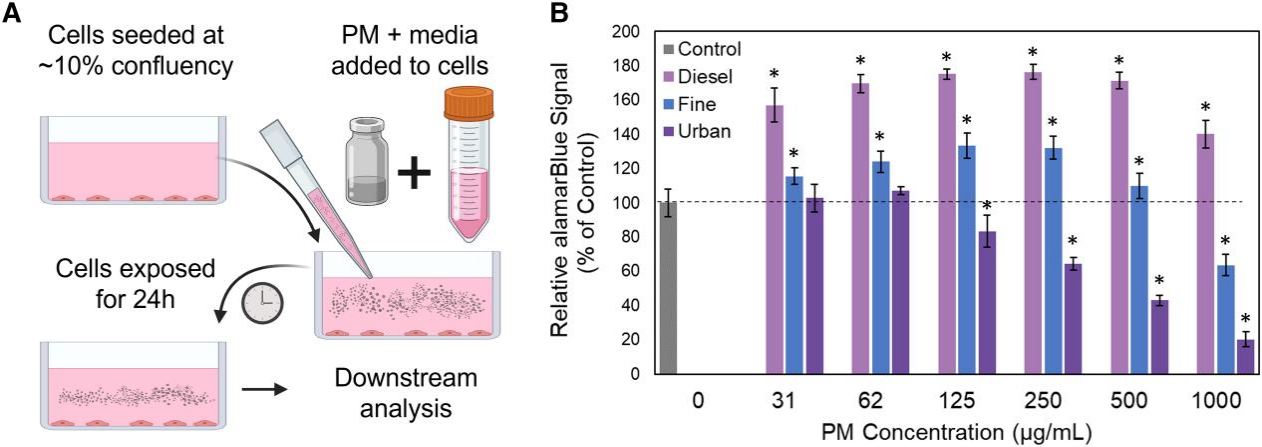
Effects of PM exposure on cell viability. A) Graphical depiction of submerged PM exposure method. Created with BioRender.com. B) Cell viability following 24 h exposures to different PM types and concentrations. Values are percentages of viable cells relative to unexposed control cells as measured with the alamarBlue assay (*n* = 7, error bars represent 1 SD, *P < 0.05 using Student's t-test).

To evaluate the effects of PM exposures on cellular viability, we used the alamarBlue assay which measures the reductive capacity of cells as a proxy for viability. We observed that after cells were exposed to PM for 24 h (Fig. 1A), cell populations exhibited PM-type- and concentration-dependent changes in alamarBlue signal (Fig. 1B). For example, urban PM induced a steady decrease in signal at concentrations ≥250 µg/mL (*P* < 0.05). However, fine PM induced a significant decrease in signal only at the highest concentration of 1,000 µg/mL. Paradoxically, diesel exhaust PM induced an increase in signal across all concentrations, as shown previously (39). Often interpreted as an increase in viability, however, the increase in signal could be associated with metabolic change in the capacity of cells to reduce resazurin in the alamarBlue. This prompted us to look further into this increase by evaluating the change in alamarBlue signal at short times. A 1.5 h exposure to diesel PM showed similar increases, indicating the cellular metabolism of the exogenous PAHs in this PM sample may be increasing the alamarBlue signal as a significant change in the number of cells would not occur at this shorter time point (Fig. S1). It should be noted that we also tested if the diesel PM was directly interfering by reducing the resazurin in the alamarBlue assay; for these experiments, we incubated alamarBlue with cell media with and without the diesel PM, and we saw no difference in signal (Fig. S1).

The exposure concentrations of 125 and 500 µg/mL, equivalent to 35.2 and 140.8 µg/cm^2^ in terms of deposition over cell growth area, were chosen for further analysis. These concentrations were chosen based on previous analyses indicating that 20 µg/cm^2^ could be deposited in the tracheobronchial regions of the lung over a period of 8 h in an urban environment (40), ∼35.2 µg/cm^2^ falls within an expected deposition amount within areas of the human lung for a 24-h period in an urban environment, and ∼140.8 µg/cm^2^ could be representative of exposure levels in extremely polluted cities.

### Exposure to different PM mixtures induces differential DNA damage responses and cell death

**Fig. 2.**
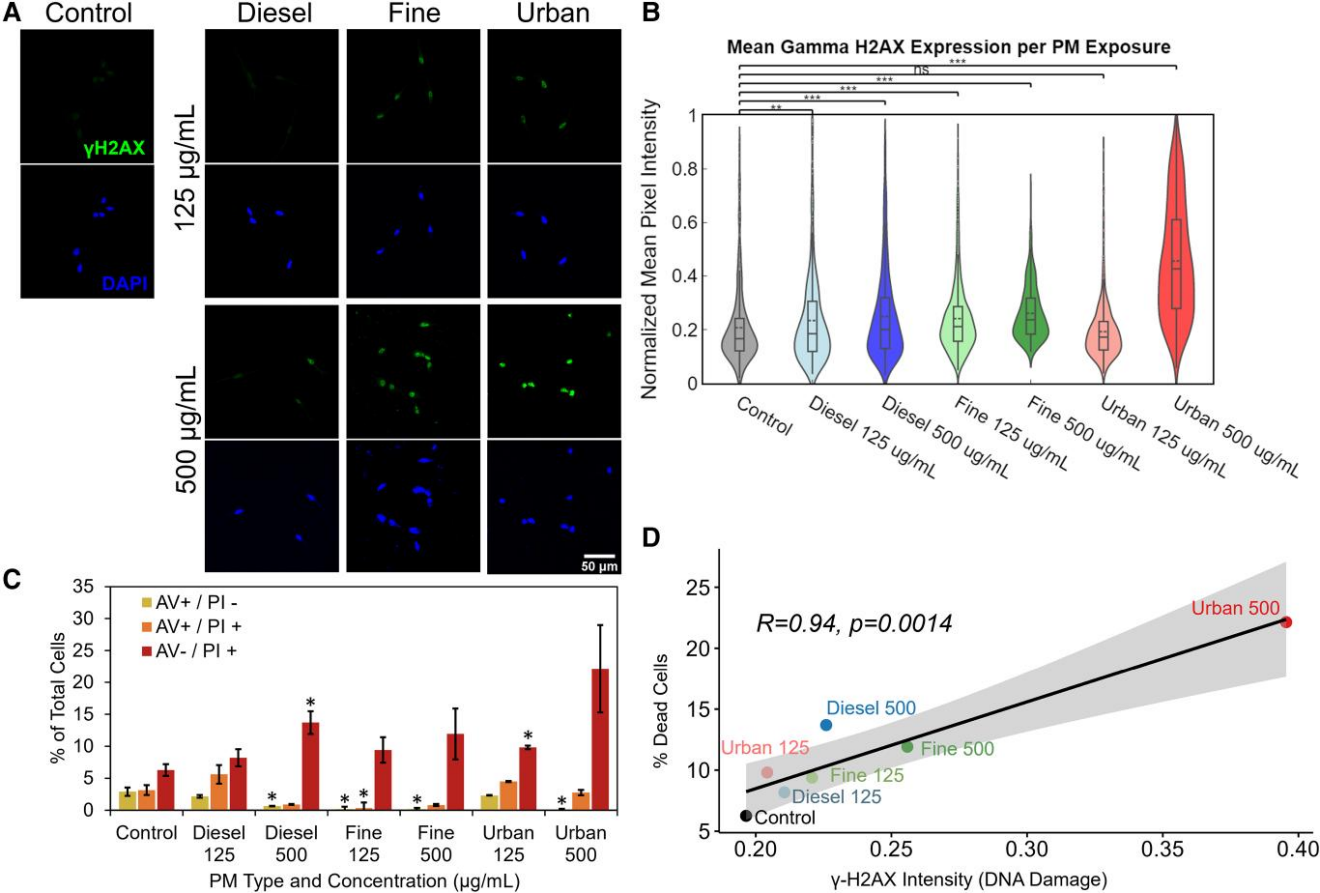
PM exposure leads to alteration of apoptotic levels and DNA damage. A) Representative images of the immunofluorescent staining of γH2AX across different exposure conditions. B) Violin plots overlaid with box and whisker plots showing the distribution of average γH2AX intensity values for cell nuclei in each exposure condition. C) Flow cytometry analysis of AV–PI apoptosis assay following PM exposure. AV+/PI– and AV+/PI+ represent early- and late-stage apoptotic cells, respectively, AV–/PI+ represents dead cells (*n* = 3, ≥10,000 cells per measurement, error bars represent the standard error of the mean, *P < 0.05, comparisons are drawn against the respective control populations using Student's t-test). The remaining cells in each condition were healthy (AV–/PI–). D) Correlation between the percentage of dead cells from the apoptosis assay shown in (A) and the average γH2AX intensity following PM exposure. Shading represents a 95% CI.

Interestingly, the alamarBlue assay showed PM-dependent changes in signal across a wide range of concentrations (up to 500 µg/mL). To investigate this further, we profiled the DNA damage responses to PM mixtures. Using confocal microscopy, we measured the accumulation of the histone phosphorylation γH2AX as a marker of double stranded DNA breaks, a marker of genotoxicity and cell death (41). We found that exposures to the different PM mixtures at concentrations of 125 or 500 µg/mL led to differential levels of DNA damage, with exposures leading to increases in DNA damage relative to the control, unexposed cells (Fig. 2A and B). Additionally, at higher exposure concentrations, γH2AX staining in some cells becomes pan-nuclear which is indicative of apoptosis in other cell types (Figs. 2A and S2) (42, 43).

Furthermore, we evaluated whether PM exposures were inducing cell death via apoptosis or based on nonapoptotic mechanisms. Both apoptotic and nonapoptotic mechanisms are associated with aberrant levels of DNA damage. To determine the mode of cell death, we used an annexin V (AV)–propidium iodide (PI) flow cytometry assay, as previously used to investigate the mode of cell death in lung cells exposed to PM mixtures (44).

Cells exposed to 125 µg/mL of all PM conditions exhibited small increases in the population of dead cells (AV–/PI+) and a decrease in the population of apoptotic cells (AV+/PI– and AV+/PI+; Fig. 2C). AV+/PI– indicated cells were in the early stages of apoptosis, while AV+/PI+ indicated cells are in later stages of apoptosis or dying due to loss of membrane integrity. In contrast, exposure to 500 µg/mL concentration of each PM mixture resulted in an increase in the number of dead cells (AV–/PI+; Fig. 2C). Representative scatter plots of flow cytometry data from each condition are shown in Fig. S3. We next compared the trends in the populations across the different PM mixtures and observed that exposure to different PM compositions led to different distributions of apoptotic vs. dead cells. For instance, exposure to the urban PM mixtures resulted in a greater number of dead cells relative to the fine and diesel mixtures. These results corroborate the general pattern in viability observed via the alamarBlue assay, with urban PM inducing the greatest losses of viability, but better captures loss of viability in the diesel and fine conditions given the alamarBlue assay also captured increases in metabolic activity of PM components (Figs. 1B and S1).

In the literature, AV–/PI+ events are sometimes reported as cells undergoing mechanisms of death such as ferroptosis or cells lacking a cell membrane, resulting in no AV signal (45, 46). This would be consistent with our results given our collection of attached and detached cells following the 24 h length of exposure. It is therefore possible that following PM exposures, some cell membranes may have been fully ruptured, leaving nuclei behind. However, it is also possible that other forms of cell death are taking place such as ferroptosis, resulting from the exposure to iron and other metals present in the PM mixtures.

The levels of DNA damage are also associated with the levels of observed cell death (Fig. 2D). As shown in Fig. 2, γH2AX intensity increases with exposure to increasing PM concentrations for each of the three PM types. Furthermore, γH2AX intensity positively correlates (Pearson Coefficient of *R* = 0.94, *P* = 0.0014) with the percentage of dead cells (AV–/PI+) found in the AV–PI data for the same exposed populations (Fig. 2D). Taken together, these data show increases in cell death and DNA damage levels are observed with increasing PM concentrations. These levels are also dependent on the PM composition, as the three mixtures show markedly different trends. Additionally, the correlation between cell death and γH2AX intensity points to a framework of DNA damage-associated cell death.

### Post-PM exposure transcriptional profiling indicates common and unique gene expression remodeling

The unique differences observed in viability and the patterns of DNA damage following PM exposures at 125 and 500 µg/mL prompted us to investigate whether PM exposures also induced differential molecular responses. To better understand how the underlying transcriptomic profiles influence differential viability across PM mixtures, we assessed changes in gene expression patterns via 3′-TagSeq (47, 48) (Datasets S2–S7). This approach takes advantage of the poly(A) tail on mRNA for sequencing library preparation, allowing the accurate quantification of protein-coding transcripts.

**Fig. 3.**
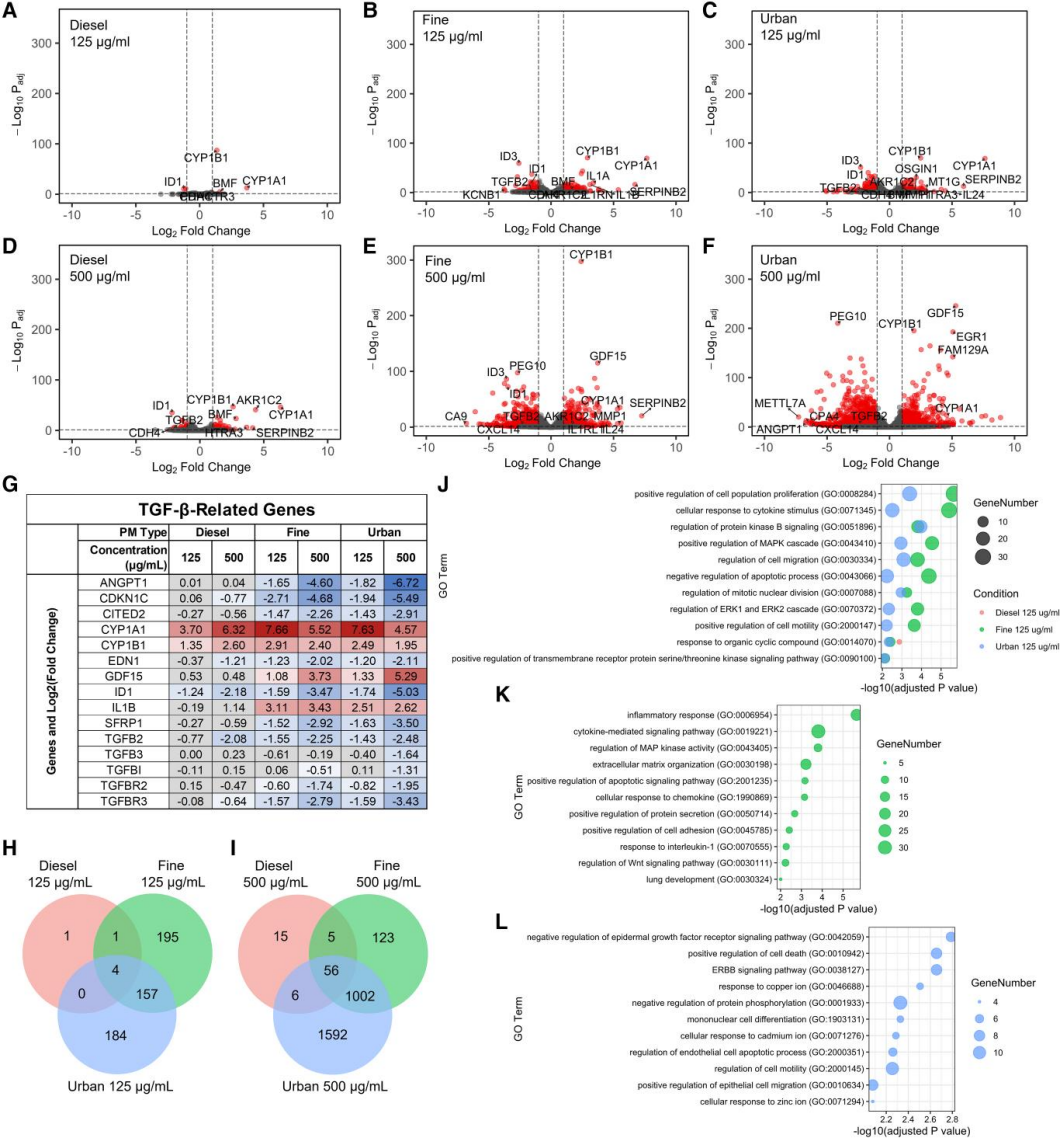
Transcriptomic analysis of PM stress reveals unique network remodeling. A–F) Volcano plots showing significantly DE genes (cutoffs = Log_2_FC > 1, *P*_adj_ < 0.05) relative to control cells after exposure to diesel, fine, and urban PM at 125 µg/mL (A–C) and 500 µg/mL (D–F) for 24 h. G) Log_2_Fold changes in expression of select TGF-β-related genes. A gray background indicates the expression change was not significant (*P* > 0.05). H and I) Venn diagrams of the significantly DE genes from each condition. Intersections represent genes that were DE in overlapping conditions. J–L) Bubble plots showing select enriched GO biological process terms that are commonly enriched among 2 or more of the low-level exposure conditions (J), or unique to the low-level fine (K) or urban (L) exposure conditions.

We first observed that exposure to urban and fine PM mixtures induced significant changes in the expression of a greater number of mRNA transcripts relative to diesel exhaust under the two PM concentrations tested. Furthermore, the magnitudes of the changes were larger for cells exposed to urban and fine mixtures than those exposed to diesel exhaust PM (Fig. 3A–F). These observations indicate that the urban and fine mixtures have a lower threshold for stimulation of cellular responses. Additionally, the number of genes that were differentially expressed (DE) by each PM type increased with higher concentrations (i.e. 125 µg/mL vs. 500 µg/mL exposures; Fig. 3A–F). Moreover, the majority of the genes (at least 67% for each condition) that were up- and down-regulated in the 125 µg/mL conditions were similarly up- and down-regulated in the 500 µg/mL condition (Fig. S4), indicating consistency in the transcriptional responses across different concentrations of each PM type, with additional pathway activation at higher concentrations.

Additionally, we observed that four mRNAs encoded by the CYP1A1, CYP1B1, ID1, and ID3 genes were DE postexposure across all conditions (Fig. 3G); two were overexpressed (CYP1A1 and CYP1B1) and two displayed decreased expression (ID1 and ID3), relative to expression levels in unexposed control cells. The CYP1A1 and CYP1B1 are members of the cytochrome P450 (CYP) family that metabolize endogenous compounds such as fatty acids and steroid hormones (49). Consistent with our results, these genes are upregulated in human epithelial lung cell models in response to exogenous PAHs present in PM (15). These PAHs bind to the cytosolic aryl hydrocarbon receptor, which then mediates expression of the cytochromes and promotes a proinflammatory response to induce reactive oxygen species (ROS) production in cells. ID1 and ID3 are inhibitors of DNA binding proteins that are induced by transforming growth factor β (TGF-β) and have been implicated in the regulation of senescence, apoptosis, and cell cycle alterations (50). Moreover, ID1 expression has also been shown to decrease after exposure to coarse PM (PM with an aerodynamic diameter between 2.5 and 10 µm) (11), but the roles of these genes have been less defined in the context of air pollution exposures. Importantly, the increase in expression of the CYP genes and the decrease in expression of the inhibitor of DNA binding (ID) genes suggest that the response to organic cyclic compounds as well as alteration of the TGF-β regulatory pathways is commonly remodeled by these unique PM mixtures. This is further supported by the differential expression of additional TGF-related genes (Fig. 3G). Interestingly, many TGF-β-related genes are involved in the regulation of cell morphology and motility.

We next observed that unique mRNAs were significantly DE only when cells were exposed to certain PM mixtures, but not others (Fig. 3H and I). For example, TNFAIP6, a regulator of the ECM, LCAT, a protein involved in extracellular metabolism, and CXCL1, a protein involved in inflammation, are significantly DE under only fine exposure conditions. However, genes including DDIT4, a protein induced by DNA damage, MT1E, a protein involved in the cellular response to cadmium, and ACTN4, an actin-binding protein, are DE under only urban exposure conditions. This indicates that there could be unique pathway activation that is dependent upon the PM composition.

Overall, the gene expression patterns observed in cells exposed to fine and urban PM mixtures exhibit significant pathway remodeling, whereas cells exposed to diesel exhaust PM exhibit less remodeling. Similarly, we observed a dose dependence in the extent of pathway remodeling, i.e. more changes with higher PM concentrations. Finally, we noted that although expression of a limited set of 4 genes was consistent across all conditions (i.e. CYP1A1, CYP1B1, ID, and ID3), other genes are DE in a manner that is dependent on the PM type.

### Gene ontology analysis reveals PM-dependent remodeling of apoptosis, motility, and morphology pathways

To determine the key remodeled pathways postexposure and the extent to which they were remodeled, we performed Gene Ontology (GO) and pathway enrichment analysis. We performed this analysis using the transcriptomics data from cells exposed to urban, fine, and diesel exhaust PM mixtures at 125 and 500 µg/mL (Figs. 3J–L and S5). The complete list of enriched GO terms for each condition is given in Datasets S8–S13. Using Enrichr (51), we identified 34 pathways that were significantly enriched (*P*_adj_ < 0.01) in cells exposed to the 125 µg/mL concentration of both urban and fine PM mixtures, relative to baseline. We selected 11 nonredundant pathways to show in Fig. 3J. We observed changes in the expression of genes related to the mitogen-activated protein kinase (MAPK) cascade (e.g. EDN1, GDF15, TGFB2, ANGPT1, and LIF), epithelial cell proliferation (e.g. CDKN1C and EPGN), regulation of apoptosis (e.g. FCMR and CITED2), and cell migration and ECM organization pathways (e.g. SFRP1 and FGG). It is worth noting that for cells exposed to urban and fine PM at 500 µg/mL, similar pathways were also significantly enriched (*P*_adj_ < 0.01; Fig. S5 and Datasets S11–S13).

Interestingly, we observed few DE genes in cells exposed to the diesel exhaust PM mixture. Only the “response to organic cyclic compound” pathway was significantly enriched (*P*_adj_ < 0.01) in cells exposed to all PM types at the 125 µg/mL concentration. However, this response appears to be ubiquitous, with the CYP1A1 and CYP1B1 genes increasing in expression across all conditions postexposure. Similarly, we identified upregulation of IL1B, which was upregulated in all conditions except 125 µg/mL diesel exhaust PM.

We also identified key genes exhibiting differential expression across both the 125 µg/mL urban and fine PM exposure conditions that contributed to the remodeling of multiple pathways (Figs. 3I–J and S6). For example, IL1A, IL1B, and TGF-β genes were part of several GO-defined pathways that comprise cytokine signaling cascade and TGF-β signaling. Other genes involved across many pathways include GAS6, which is involved in cell growth and migration and cytokine signaling, and PTK2B, a protein involved in the activation of MAPK signaling and reorganization of the actin cytoskeleton. These genes are present in many of the most significantly altered pathways, highlighting their importance in the biological response to PM exposure.

Finally, we observed that several exclusive GO terms were significantly enriched (*P*_adj_ < 0.01) in cells postexposure to urban PM at both concentrations (125 and 500 µg/mL; Figs. 3K and S5), which included unique responses to metal ions. Examples of these pathways include response to cadmium ion, copper ion, and zinc ion, which encompass mRNAs encoded by the MT1 family genes (MT1G, MT1E, MT1F, and MT1M). The patterns of gene expression changes involved in the regulation of metal ions are consistent with the increase in metal composition (i.e. cadmium) in the urban PM mixture, relative to the other mixtures tested (Table 1). Similar to the 125 µg/mL urban exposure, at the 500 µg/mL urban condition, the top significantly enriched GO term is a response to the metal ion, again indicating the importance of the increased metal concentrations in the urban PM sample relative to fine and diesel exhaust.

Taken together, these data indicate that cells differentially regulate their gene expression patterns in a PM composition-dependent manner. However, pathways related to cell morphology, and ECM remodeling seem to be broadly shared across all PM exposure conditions, with pathways related to apoptosis shared across the urban and fine conditions.

### PM compositions drive the emergence of morphological subtypes postexposure

**Fig. 4.**
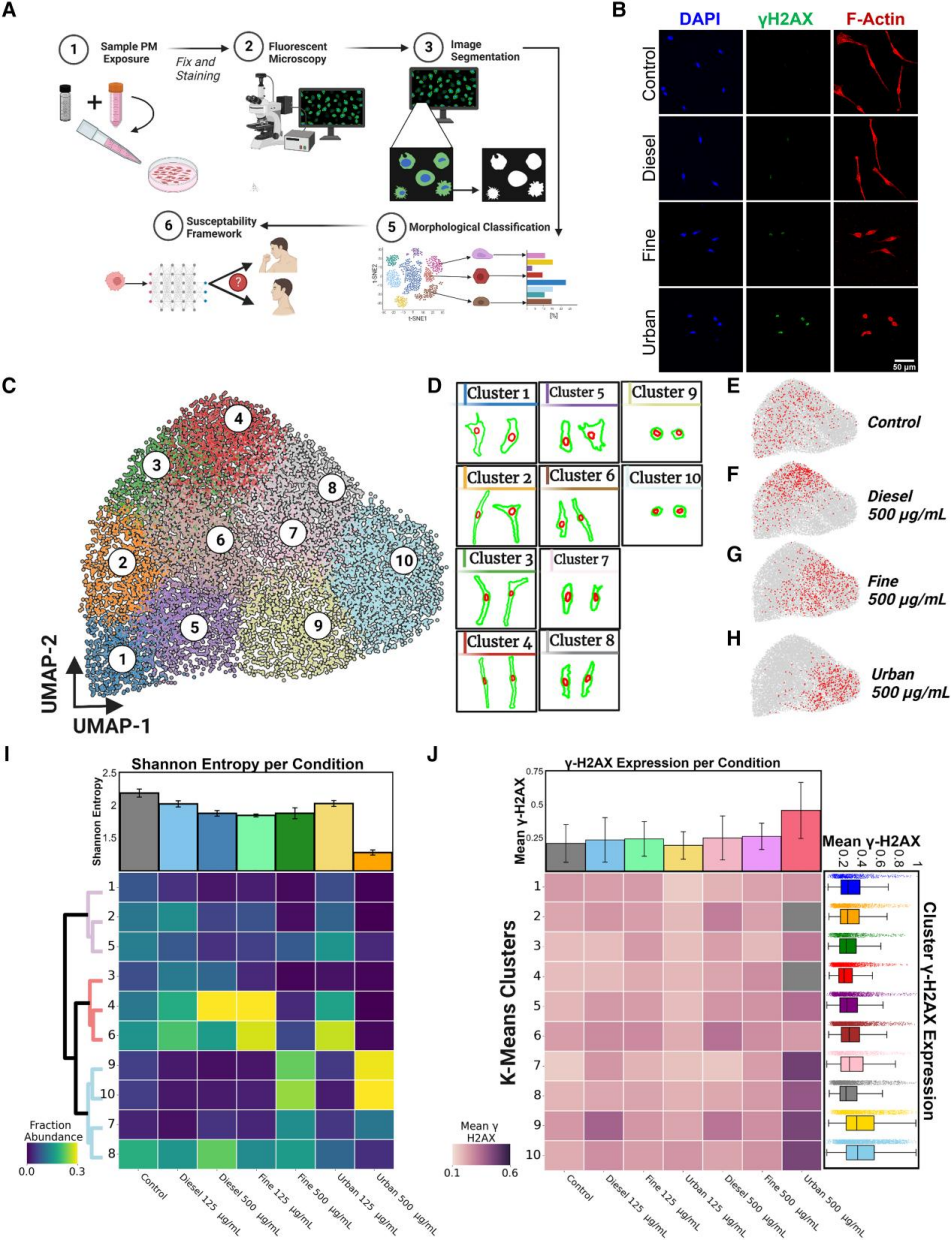
Morphological analysis reveals distinct cellular subtypes. A) Graphical depiction of the morphological analysis pipeline. B) Representative fluorescence microscopy images of cells from each condition. C) UMAP visualization of the 33 measured morphological parameters for each cell in every condition. UMAP-1 (*x*-axis) was *negatively* correlated with size and UMAP-2 (*y*-axis) was *positively* correlated with cell elongation, or linearity. *k*-means clustering was applied to cluster cells of similar morphologies. D) Representative cellular (outer) and nuclear (inner) morphologies of cells from each *k*-means morphology cluster. E–H) Plots showing the distribution of cells from each respective exposure condition in red within the UMAP space. I) Heatmap displaying the enrichment in a number of cells in each morphology cluster for each exposure condition. The bar graph shows the Shannon entropy for the distribution of cell morphologies within each exposure group. The dendrogram identifies clusters with similar morphological features. J) Heatmap displaying the mean γH2AX in morphology clusters across all exposure conditions. Mean γH2AX intensity across each exposure condition (top). γH2AX intensity of all cells within each *k*-means cluster (right). Panel A created with BioRender.com.

Since unique PM mixtures drive differential responses, particularly in apoptosis, cytoskeletal structure, and ECM-related pathways, we wondered whether these responses could be captured by changes in cellular morphologies across cell populations. Using our BEAS-2B cell line model, we exposed cells to the same PM mixtures at the same concentrations and exposure times described above. After exposure, cells were fixed and stained for F-Actin (488-Phalloidin), DNA (DAPI), and γH2AX (anti-γH2AX [phospho-S139] antibody; Fig. 4A and B). The Phalloidin and DAPI stains were used to delineate the cell and nuclear boundaries, and γH2AX to quantify the extent of persistent DNA damage. For each cell and nuclear boundary, we computed 33 discrete parameters describing features related to the sizes and shapes of individual cells (Table S1). Across all conditions, we analyzed ∼13,000 single cells. To identify whether BEAS-2B cells exhibited morphological subtypes that changed after PM exposure, we performed dimensional reduction and clustering analyses on cells analyzed across all conditions. Using a combination of *k*-means clustering and uniform manifold approximation and projection (UMAP), we identified 10 distinct morphology clusters, each having unique cellular and nuclear morphological profiles (Fig. 4C and D). Furthermore, these 10 morphological clusters can be further grouped into 3 cluster groups (CGs), with morphology clusters 1, 2, and 5 belonging to CG1, morphology clusters 3, 4, and 6, belonging to CG2, and morphology clusters 7–10 belonging to CG3 (Fig. S7).

Next, we asked whether cells exposed to both low (125 µg/mL) and high (500 µg/mL) concentrations of each PM mixture exhibited differential abundance of cells across each morphology cluster. Upon comparison, we observed pronounced shifts in the abundance of cells per morphology cluster in a PM-dependent manner (Figs. 4E–I and S8). Specifically, when compared with unexposed conditions, cells exposed to 500 µg/mL of urban and fine PM exhibited higher fractions of cells in clusters 9 and 10, which describe smaller, more rounded morphologies (Fig. 4G–I). However, cells exposed to 500 µg/mL of diesel PM exhibited higher fractions of cells in clusters 4 and 8, which describe larger, more elongated cell morphologies (Fig. 4F–I). Based on the observed fractional redistributions among morphology clusters per condition, we computed the Shannon entropy as a way to estimate cellular heterogeneity (52). Although cells were redistributed among morphology clusters per PM conditions, only cells exposed to urban 500 µg/mL showed a pronounced decrease in heterogeneity relative to unexposed control cells (Fig. 4I).

Taken together, our results indicate that cells exposed to different PM mixtures drive fractional redistributions among cellular morphology clusters in a PM-dependent manner. Furthermore, the differential shift in localization of cells exposed to urban PM (toward small, more rounded morphologies, especially at the 500 µg/mL exposure concentration) and diesel PM (toward larger, more elongated morphologies) point out that these PM mixtures are likely driving unique responses based on the underlying compositions. Finally, these results suggest the potential utility of cell morphology cluster profiles to denote functional subtypes in pre- and postexposed cells.

### Morphological clusters are characterized by the extent of persistent DNA damage

Given that cells exposed to both urban and fine PM exhibited a higher fraction of cells with smaller, more rounded cell morphologies (Fig. 4G and H) and decreased viability relative to unexposed cells (Fig. 1B), we investigated whether morphology clusters were associated with persistent DNA damage. Since each cell was costained for γH2AX, we computed the extent of DNA damage based on the total nuclear abundance of phosphorylated-H2AX (γH2AX) as previously performed (53). Comparing cells from all exposure conditions, we observed a significant increase in the γH2AX content for cells exposed to 500 µg/mL urban PM relative to unexposed control cells. Additionally, viewing the cells at higher magnification shows an increase in the number of γH2AX foci following exposures (Fig. S2). Furthermore, to test whether cells in different morphology clusters exhibited different levels of DNA damage, we pooled cells within each morphology cluster across all conditions and quantified the levels of γH2AX. Interestingly, we found that cells belonging to clusters 9 and 10 had the highest levels of γH2AX (i.e. high DNA damage), with cluster 4 exhibiting the lowest level of damage (Fig. 4J). These results suggest that the identified cell morphology clusters could be further defined based on the extent of DNA damage and susceptibility to cell death after PM exposure.

### Cadmium drives morphological shifts among functional clusters after PM exposures

**Fig. 5.**
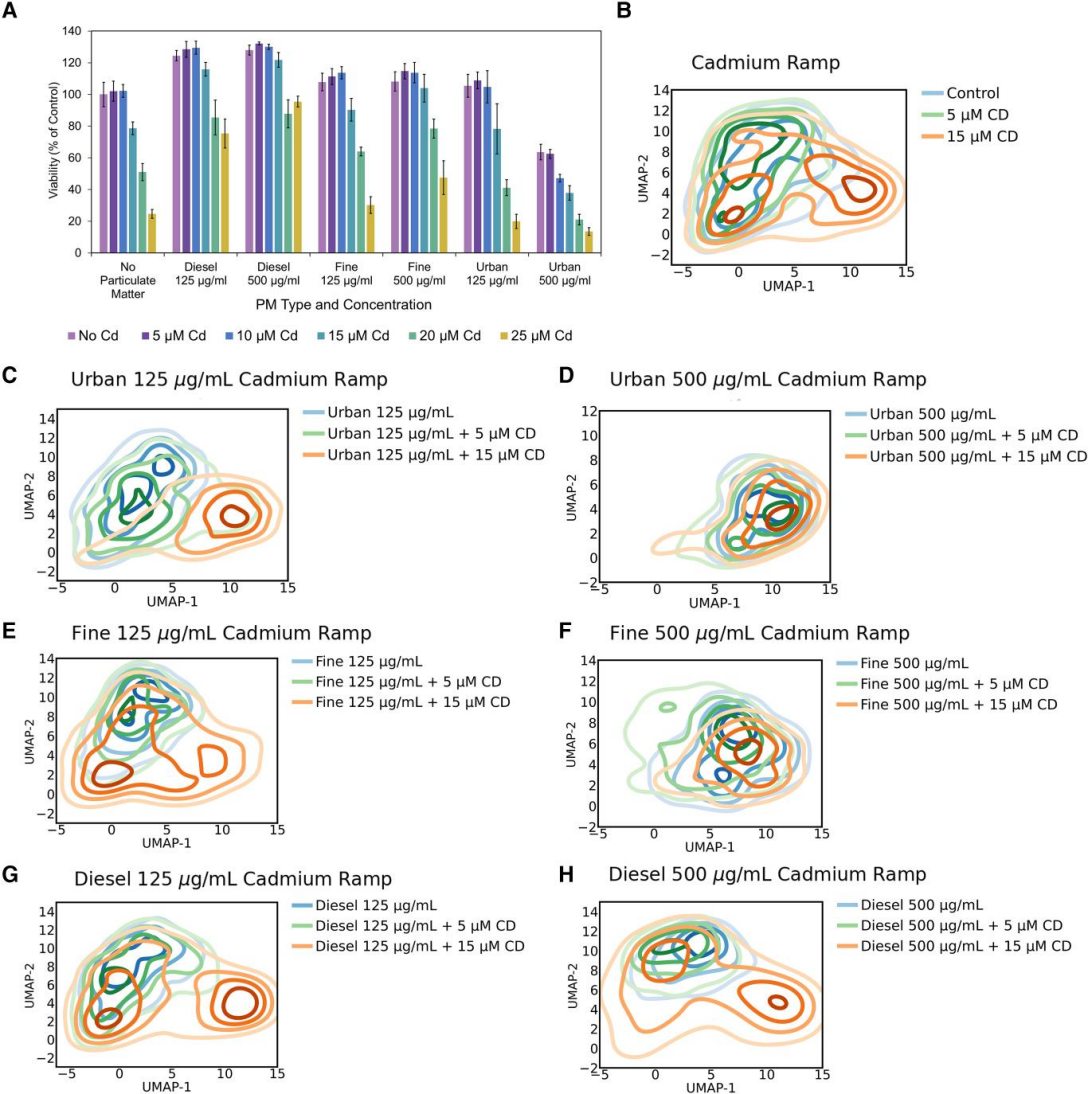
Cadmium drives morphological shifts in cells. A) Cell viability following 24 h exposures to different PM types and concentrations supplemented with cadmium chloride (0–25 µM Cd). Values are percentages of viable cells relative to unexposed control cells as measured with the alamarBlue assay (*n* = 6, error bars represent 1 SD). B–H) Morphological distributions of cells from each respective exposure condition with CdCl_2_ supplementation displayed across the UMAP space.

To further test the hypothesis that the chemical compositions of the PM mixtures drive specific shifts among morphological clusters (i.e. smaller, rounder, and less viable cells), we systematically supplemented our PM mixtures with different concentrations of cadmium chloride (0–25 µM) and lead acetate (0–250 µM) that mimic those used in other studies (54, 55). We selected cadmium (Cd) and lead (Pb), due to their variable concentrations across different PM mixtures (Table 1), and the pronounced shifts in both the viability and the morphological shifts when cells were treated with urban PM (urban PM has the highest concentration of Cd in the tested PM mixtures). First, we observed a significant decrease (*P* ≤ 0.05) in viability with increasing levels of cadmium chloride supplementation across all conditions tested (Figs. 5A and S9). In contrast, cells exposed to PM mixtures supplemented with lead acetate resulted in little to no change in viability (Fig. S10).

Evaluating the morphological effects of cells exposed to increasing CdCl_2_ concentrations across all PM mixtures (Fig. 5B–H), we observed a general tendency toward smaller, rounded morphologies described by clusters 9 and 10. Cells exposed to 125 µg/mL urban PM and 15 µM Cd resembled the distributions of 500 µg/mL urban either alone or with 5 or 15 µM cadmium supplementation (Figs. 5C, D and S11B, C). For cells exposed to 125 µg/mL of fine PM, 15 µM cadmium supplementation led to a great shift relative to the 5 µM. However, in the 500 µg/mL fine PM conditions, even at 5 µM we observed a shift toward clusters 9 and 10, with 500 µg/mL fine PM with cadmium supplementation resembling the 500 µg/mL urban PM conditions (Figs. 5E, F and S11D, E). Finally, cells exposed to 125 µg/mL diesel PM with 15 µM cadmium exhibited a bi-phasic shift in the abundance of cells among clusters, with 33.9% of cells shifting toward clusters 9 and 10. However, cells exposed to 500 µg/mL of diesel and 15 µM cadmium exhibited a similar shift toward clusters 9 and 10 (30.4% of cells), despite the increased PM concentration (Fig. 5G and H).

Collectively, our data indicate that the differential abundance of cadmium in the different PM mixtures may drive differential toxicity among PM mixtures. Importantly, these observed correlations between increased PM toxicity (lower viabilities with cadmium supplementation) and distinct morphological redistributions among cell populations suggest the potential for predicting the toxicity and susceptibility of cells to different PM mixtures using their morphologies.

### Single-cell morphology is associated with cellular susceptibility to urban PM exposures

**Fig. 6.**
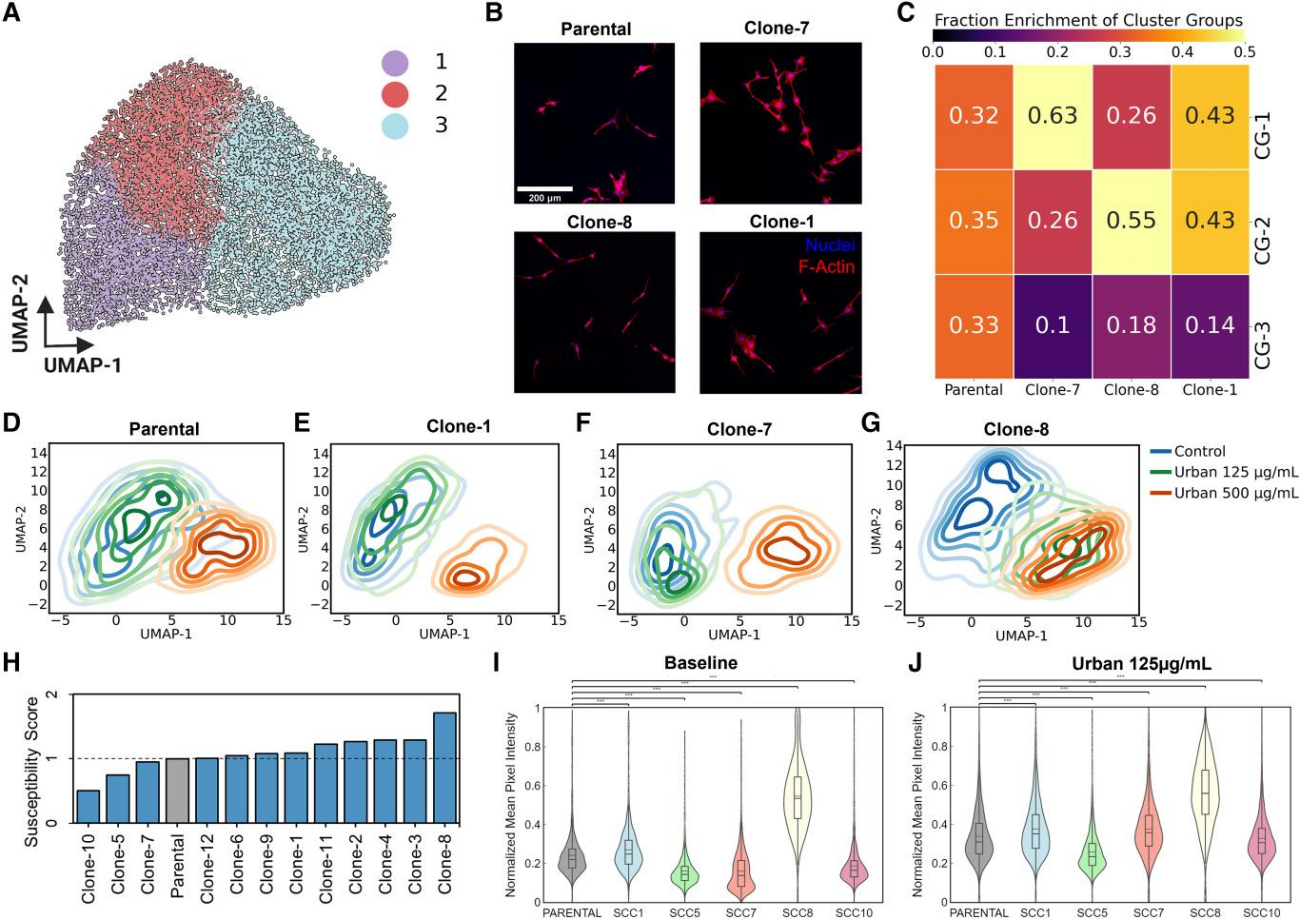
Morphology encodes susceptibility to PM exposure. A) The 10 *k*-means clusters that are used to define cell morphology can be further grouped into 3 CGs (CG1–3) using hierarchical clustering. B) Representative images of each population. C) Clonal populations show enrichment in different morphological CGs. The distributions of cell morphologies differ for each clone and the parent cell population from which the clones were derived. D–G) Upon exposure to urban PM, clones with unique baseline morphologies show different shifts in morphology. H) Susceptibility scores for each cell population normalized to the Parental population. A more positive value indicates a higher susceptibility to PM stress. I and J) Average γH2AX intensity values in cell nuclei for populations in the baseline and urban 125 µg/mL exposure condition. Significance determined by one-way ANOVA test, ***P < 0.001.

Since cells exhibited unique morphologies and responses to PM exposures, we wondered whether cellular morphologies encoded resilience or reduced susceptibility to PM exposure at the single-cell level. To test whether the morphologies of cells were associated with the response to PM exposure, we isolated single-cell clones from the parental BEAS-2B cell line. Seeding a single cell per well of a 96-well plate, we generated 12 single-cell clones. Analyzing the morphologies of each clone, we did not observe any clone localizing specifically to one morphology cluster (Fig. 4C). However, when separating the individual morphological clusters into the three CGs (CG1, CG2, and CG3), we observed that some clones differentially occupied one or multiple of the three CGs (Figs. 6A–C, S12A, and S13). As expected, when we compared the cellular heterogeneities (i.e. cell-to-cell variations) of the 12 clones relative to the parental, we observed an overall reduction in the Shannon entropy for each of the clones, with clone 7 having the lowest heterogeneity (Fig. S14).

To further test the hypothesis that cellular morphologies governed the response to PM mixtures, we exposed all clones to the urban PM mixture at 125 and 500 µg/mL for 24 h. To illustrate unique baseline morphologies, we selected the parental and three clones that exhibited differential abundance of cells within the three morphological CGs. Specifically, at baseline the parental line had a similar abundance of cells across all three CGs, clone 7 was highly abundant for cells in CG1, clone 8 was highly abundant in CG2, and clone 1 was highly and equally abundant in CG1 and CG2 (Fig. 6C). Based on these starting morphologies, we tested the cellular responses to urban PM exposures (both 125 and 500 µg/mL).

After exposure, single-cell clones showed differences in morphological distributions. For the parental and isolated clonal populations, cells exposed to 500 µg/mL of urban PM resulted in a drastic shift toward CG3 and more specifically morphology clusters 9 and 10. However, the major differences in morphological shifts were observed in the cells exposed to 125 µg/mL of urban PM (Figs. 6D–G and S12B). For the parental and clone 7 populations, there were very little shifts in the abundance of cells within the CGs, as shown by the significant overlap of contours from control (unexposed) and the 125 µg/mL conditions (Fig. 6D and F). Interestingly, for clone 8, at 125 µg/mL urban PM, there was a significant shift toward CG3 (specifically clusters 9 and 10), while clone 1 showed a shift toward CG2, the intermediate morphological regime (Fig. 6E and G). These results suggest that clone 8 may be more susceptible to urban PM relative to clone 7, while clone 1 is moderately susceptible. We proposed the shifts in morphology (i.e. morphological transitions rather than baseline morphologies) captured by the UMAP space could be used to quantify cell population susceptibility to PM exposure using the following equation:


(1) $$S_{\mathrm{SC}}=\frac{\left({\mathrm{CG}}_{1}^{\mathrm{UL}}-{\mathrm{CG}}_{1}^{C})-({\mathrm{CG}}_{1}^{\mathrm{UH}}-{\mathrm{CG}}_{1}^{\mathrm{UL}}\right)}{\left({\mathrm{CG}}_{1}^{\mathrm{UH}}-{\mathrm{CG}}_{1}^{C}\right)}$$

where *S*_SC_ is the susceptibility score, CG_1_ represents the fraction of cells within cluster group 1 at each respective condition (C = control, UL = urban 125 µg/mL, and UH = urban 500 µg/mL). This equation quantifies the shift away from the healthy CG1 morphology, giving higher scores when populations shift away from CG1 at the lower exposure concentration. The resulting scores (Fig. 6H) show a spectrum of susceptibilities with clone 8 being the most susceptible, followed by clones 2, 3, and 4 which showed more drastic morphological shifts at the 125 µg/mL exposure condition. Additionally, clones 10 and 5 had the lowest susceptibility scores.

To further test this notion of susceptibility based on morphological shifts, we evaluated whether DNA damage responses followed our susceptibility scoring (Fig. 6I and J). Clones 5, 7, and 10 showed low expression of γH2AX at baseline, and clones 5 and 10 continued to show the lowest levels following exposure to 125 µg/mL of urban PM, agreeing with the susceptibility scores. Additionally, clone 1 was ranked as more susceptible than the parental population and showed significantly higher expression of γH2AX. Additionally, clone 8 exhibited the highest γH2AX signal at baseline (>2× compared to the parental, *P* < 0.001), with a significant increase at 125 µg/mL of urban PM (*P* < 0.001).

Taken together, these results point to the notion that quantifiable shifts in cell morphology profiles can be used as predictors or biomarkers of PM-induced responses, even at the 125 µg/mL concentration which did not induce drastic increases in cell death (Fig. 2C). Clones with a high abundance of cells in CG1 and lower numbers of cells shifting to CG2 or CG3 upon exposure were most resilient to urban PM, while clones with large shifts toward CG2 and CG3 demonstrated increasing susceptibility to urban PM. Finally, susceptibility to urban PM exposures seems to be influenced by the levels of DNA damage.

## Discussion

In this study, we demonstrate a multiscale approach to characterize the unique differences in cellular response to three PM mixtures using molecular and quantitative morphological analyses. We further investigated morphological variations across populations of unexposed and PM-exposed cells to show that cellular morphology is associated with susceptibility to urban PM exposures and provides mechanistic insights into variable responses across cell populations. Additionally, we show that these responses are dependent on the composition of the PM mixture, for instance, an abundance of cadmium can drive unique cellular transcriptional responses and morphological changes.

**Fig. 7.**
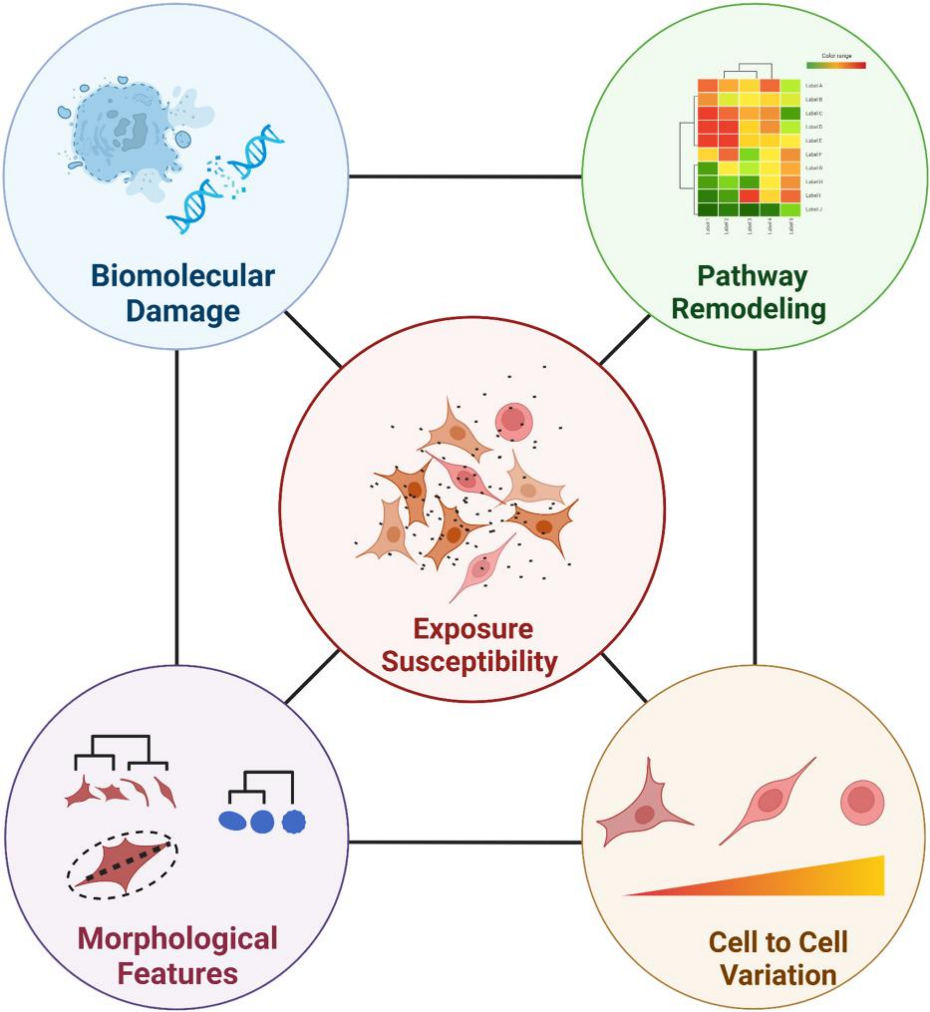
Model of exposure susceptibility. Cellular morphology encodes susceptibility to PM exposure and is dependent upon the interplay between molecular changes in cells. Created with BioRender.com.

With the emergence of single-cell technologies and deep-learning tools, there has been a tremendous acceleration in the capacity to quantify and analyze specific cell states and behaviors across cell populations (56–58). Specifically, analysis of biophysical properties, such as motility and morphology, offers an efficient method to discretize functional subtypes of cells (33, 59, 60). In this work, we profile the single-cell morphological changes after exposure to various PM mixtures to quantify cellular responses and identify cellular properties that are associated with cellular susceptibility to pollutants. Three major and novel findings of this work include the following: First, we identified that although there is a common transcriptomic response to PM in the activation of the P450 family cytochromes, as shown previously (15, 61), the degree of pathway remodeling is dependent on the PM composition and concentration of exposure. Second, we show cell morphology is a strong indicator of response to differential PM exposure. Third, we used single-cell clones to show that shifts from unique starting morphologies can encode susceptibility to air pollution exposure. Collectively, our findings show that cell morphology has the potential to be used as a biomarker for environmental risk assessment (Fig. 7).

With the development of single-cell technologies, in both transcriptional and morphological profiling, and the advances in RNA fluorescence in situ hybridization techniques, further studies could be performed to more directly link the expression of different transcripts with morphological features of individual cells. Additionally, the use of primary cells in a more realistic ECM environment or the use of ex vivo lung model technologies (62, 63), and the testing of additional freshly collected PM mixtures containing volatile organic components could further improve the biological context of future work. Finally, the use of live-cell imaging to monitor cellular changes over time could lead to a better understanding of cluster stability among morphological patterns and would further enhance our ability to determine susceptibility to PM mixtures and other bioactive agents.

Taken together, our data begin to elucidate how different PM mixtures drive unique changes in morphological and transcriptional signatures, and how individual cells within a population have differing levels of susceptibility, encoded in their morphologies. This knowledge could provide a better understanding of how components of PM such as cadmium and other metals drive PM toxicity. Furthermore, our platform for quantifying susceptibility could provide a potential way to investigate the effects of sensitizers and desensitizers that alter cellular responses to environmental stress. Finally, our findings could facilitate the development of morphology-based methods for characterizing an individual's risk of air pollution exposure.

## Materials and methods

### Cell culture

BEAS-2B cells (ATCC CRL-9609) were cultured from cryopreserved stocks in collagen-coated T-75 culture flasks according to ATCC guidelines. Briefly, cells were seeded at 3,000 cells/cm^2^ and cultured in 23 mL of bronchial epithelial cell growth medium (BEGM) (Lonza, CC-3170), omitting the addition of the gentamicin–amphotericin aliquot to the medium, as recommended by ATCC. Cells were grown at 37°C in a humidified incubator with a 5% CO_2_ atmosphere, and complete media exchanges were performed every 48 h. After approximately 4 days, the cultures reached ∼70% confluency, and cells were subcultured into 6-well or 96-well plates coated with type 1 collagen (Advanced BioMatrix, Cat#5005) at 1,500 cells/cm^2^, and allowed to attach to the growth surface for 24 h before exposure to PM.

### PM exposure

The BEAS-2B cells were exposed to three PM mixtures collected from different sources that were purchased from NIST, urban PM (SRM 1648a), fine atmospheric PM (SRM 2786), and diesel exhaust PM (SRM 2975). Just before the start of the exposures, the three PM mixtures were weighed using an analytical balance and suspended in sterile DI H_2_O in 10 mg/mL stock solutions. The suspensions were sterilized by ultraviolet (UV) irradiation for 30 min as done previously (44). Serial dilutions were performed with BEGM medium to reach the tested concentrations between 1,000 and 31 µg/mL. For exposures that contained supplements of cadmium, cadmium chloride was dissolved in DI H_2_O and filter sterilized before being added to the PM mixtures at 1,000× dilutions. To begin the exposures, media from the well plates was removed and replaced with equal volumes of PM-containing media for exposed cells or fresh media for unexposed control cells. The cells were incubated at 37°C in a humidified incubator with a 5% CO_2_ atmosphere for a 24-h exposure period before downstream analysis.

### AlamarBlue assay

BEAS-2B cells were seeded in 96-well plates at a density of 10,000 cells/well. Twenty-four hours later, the cells were exposed to PM mixtures at concentrations ranging from 31 to 1,000 µg/mL as described above with *n* = 7 replicates per condition. Following a 24-h exposure period, the media containing the PM was removed and 100 µL of fresh BEGM containing 10% alamarBlue (Invitrogen, DAL1025) by volume was added to each well. Cells were then incubated at 37°C for 2 h in the dark. Following this incubation, the fluorescence of each well was measured (Ex. 560/Em. 590) using a BioTek Cytation3 microplate reader. The fluorescence readouts correspond to cell metabolic activity and were normalized to the readings from unexposed control cells after performing background correction by subtracting the fluorescence of wells containing only the alamarBlue-BEGM mixture.

### AV–PI flow cytometry

In this assay, levels of the fluorescein isothiocyanate (FITC)-labeled AV protein indicate apoptosis as the AV protein binds with high affinity to the phosphatidylserine that is translocated from the inner side of the cell membrane to the outer side. Likewise, levels of PI, which fluoresces upon binding DNA in cells that have ruptured or become permeable, indicate cell death or cells that are in the latest stages of apoptosis (46, 64). The preparation of cells for flow cytometry was conducted according to established protocols (64). Briefly, following the completion of PM exposures using *n* = 3 replicates, culture media was collected and put on ice to recover detached cells. Adherent cells were trypsinized and combined with the collected culture media. The combined cells were washed twice with cold phosphate-buffered saline (PBS) before proceeding with AV-FITC and PI staining of 250,000 cells per sample using an eBioscience Annexin V Apoptosis Detection Kit (ThermoFisher, 88-8005-72). Prepared samples were analyzed on a Sony Biotechnology MA900 Cell Sorter available through the Center for Biomedical Research Support at UT Austin. At least 10,000 cells per replicate were analyzed for AV binding and PI incorporation.

### Cell staining and imaging

Following exposure, cells adhered to cover glass coated with type 1 collagen (Advanced BioMatrix, Cat#5005) were washed with prewarmed PBS for 5 min then fixed by incubation for 15 min at 37°C in a freshly prepared, methanol-free 4% formaldehyde solution in PBS. Cells were rinsed 3× with PBS before being permeabilized by incubation in a 0.1% Triton-X PBS solution for 4 min. Cells were again rinsed 3× with PBS and then blocked with 1% bovine serum albumin (BSA) in PBS for 20 min at room temperature. Cells were incubated with a 1:400 dilution of a recombinant anti-γH2AX (phospho-S139) antibody (Abcam, ab81299) overnight at 4°C to visualize the DNA damage biomarker. The next day, cells were washed 3× with PBS for 5 min and then incubated with a 1:250 dilution of a fluorescently tagged secondary antibody (Goat Anti-Rabbit IgG H&L [Alexa Fluor 488]; Abcam, ab150077) for 1 h at room temperature. Cells were then rinsed 3× with PBS and stained with Alexa Fluor 594 Phalloidin (Invitrogen, A12381) and Invitrogen NucBlue Fixed Cell ReadyProbes Reagent (DAPI) (Invitrogen, R37606) according to the manufacturer’s protocols to allow visualization of the F-actin structure and nuclei, respectively. Microscopy slides were then assembled using ProLong Gold Antifade Mountant (Invitrogen, P36930) and were sealed with clear nail polish. Slides were stored at 4°C until imaging.

Fluorescent images were acquired with a Leica Stellaris 5 Confocal Microscope at 20× resolution using 3 laser lines (405 Diode: DAPI Nuclear Stain; 488 Diode: Alexa Fluor 488 secondary antibody targeting γH2AX; 647 Diode: Phalloidin/Actin Stain). Individual Nuclei/Cell Boundaries were segmented with CellProfiler (65) in combination with in-house curation pipelines to ensure well-segmented single cells. Briefly, an immunofluorescence-focused segmentation algorithm used the DAPI stain to delineate the nucleus shape and the Phalloidin stain to delineate the general cell shape. Approximately, 13,000 single cells spanning all exposure conditions were procured for this work with an additional 40,000 cells analyzed for single-cell clones.

### Data processing and morphological analysis

Thirty-three key morphological parameters were extracted from each cell using a cell profiler morphological analysis pipeline and accounted for various degrees of communality as shown by a primary factor analysis (Table S1 and Fig. S15). Although certain parameters capture more variance than others, using all parameters with any degree of variance provides a synergistic approach to better map morphological heterogeneity (i.e. a parameter with lower magnitude communality may capture variance not captured by a parameter with a high communality). To compare morphological parameters of different scales to understand population variance, all morphological parameters were independently log normalized. The collection of parameters used in this study captures the ensemble of outputs of the morphological profiling module of cell profiler and was hypothesized to capture easily interpretable morphological heterogeneity in the cell population.

This “normalized” morphological parameter dataset was subsequently used to construct a 2D-uniform manifold and projection (UMAP) space (66). UMAP is a nonlinear dimensionality reduction algorithm that seeks to capture the structure of high dimensional data in a lower-dimensionality space (for this work, the 33-vector space was simplified down to 2). Each point in the UMAP space represents an individual cell whose morphological parameters have been transformed and projected onto the 2D-UMAP space. *k*-means clustering, an unsupervised clustering method, was used to identify distinct morphological groups within the cell dataset from the log-normalized dataset. An optimal number of clusters, 10, was calculated by a plateau in the inertia and silhouette values of the *k*-means algorithm which partitioned the clusters in an unsupervised manner (Fig. S16). To quantify morphological heterogeneity, the Shannon entropy for each PM exposure condition was calculated using the *k*-means clusters as follows:


(2) $$S=-\overset{10}{\underset{i=1}{\sum }}\;p_{i}\cdot \log\;(p_{i})$$

where *S* is the Shannon entropy (greater magnitude signifies a more heterogeneous population) and *p_i_* is the fraction of the population that is in morphological cluster i (59). For single-cell cloning analysis, larger morphological CGs were created to identify overarching morphological themes of the *k*-means clusters. Briefly, ward-based clustering was performed on the average morphological signature across each *k*-means cluster, and the analysis identified three morphological groups that encompassed the *k*-means clusters.

γH2AX content per cell was analyzed through the mean nuclear intensity of the fluorescent 488 channel as previously performed (53). Specifically, the summation of the pixel values (normalized to range from 0 to 1) of the 488 channel was divided by the pixel area of the encompassing nuclei. The resulting mean γH2AX expression was then layered across the UMAP manifold and analyzed per cluster. Approximately, 13,000 individual cells encompassing all PM exposures were analyzed for the morphological analysis.

### Single-cell cloning and live-cell imaging

Single cells of the BEAS-2B cell line were isolated using a Sony Biotechnology MA900 Cell Sorter available through the Center for Biomedical Research Support at UT Austin. Individual cells were sorted into a 96-well plate and allowed to proliferate. Media exchanges of BEGM were performed every 48 h. Cell populations were expanded to collagen-coated 24-well plates, 6-well plates, and finally, T-75 flasks before freezing cells to create multiple clonal populations. Clonal populations were then similarly used in experiments as the parental BEAS-2B population as described above. Approximately, 40,000 single cells spanning all clones and urban exposure conditions were analyzed.

### 3′-Tag RNA sequencing

BEAS-2B cells were cultured and exposed to PM as described above. Following the completion of 24 h exposures to the three PM types at 2 concentrations (125 and 500 µg/mL), RNA extraction was immediately performed on *n* ≥ 4 replicates by lysing cells with TRIzol Reagent (Invitrogen, 15596026). The RNA underwent DNase I treatment and was purified using a Direct-zol RNA Miniprep Kit (Zymo Research, R2052) according to manufacturer protocol. The purity of the RNA was confirmed using a Nanodrop 2000 Spectrophotometer (Thermo Scientific), and RNA concentration was determined using a Qubit 4 Fluorometer (ThermoFisher) RNA broad range assay kit (ThermoFisher, Q10210). Before library preparation, RNA quality was determined using an Agilent Bioanalyzer, and all samples used for sequencing had an RNA integrity number (RIN) score >8.80. The RNA was submitted to the University of Texas genomic sequencing and analysis facility for 3′ RNA-based library preparation and sequencing based on previously published protocols (47, 48). Libraries were quantified using the Quant-it PicoGreen dsDNA assay (ThermoFisher) and pooled equally for subsequent size selection at 350–550 bp on a 2% gel using the Blue Pippin (Sage Science). The final pools were checked for size and quality with the Bioanalyzer High Sensitivity DNA Kit (Agilent) and their concentrations were measured using the KAPA SYBR Fast qPCR kit (Roche). The pooled libraries were sequenced on a NovaSeq6000 (Illumina) and a sequencing depth of 4.5 million reads per sample was achieved with a single-end, 100-bp read length.

### Differential gene expression analysis

Following sequencing, the raw reads were preprocessed to remove adapter contamination and trim the unique molecular identifier barcodes, remove duplicates, and remove poor-quality reads. The Human Reference Genome was assembled and indexed using Homo_sapiens.GRCh38.dna.primary_assembly.fa and Homo_sapiens.GRCh38.104.gtf from Ensembl using the genomeGenerate run mode in STAR (Spliced Transcripts Alignment to a Reference) version 2.7.0d (67). The filtered reads were then aligned to the generated genome using STAR. HTSeq (68) was used to count the aligned reads in each .bam file generated by STAR. The DESeq2 package (69) was then used to quantify differential gene expression in R (70). Differential expression was determined for each PM exposure condition relative to the counts from unexposed control cells. Significantly DE genes were defined as having a log_2_(Fold Change) ≥ 1 and *P*_adj_ < 0.05. GO term analysis was performed using the Enrichr web tool (51) to determine GO Biological Process terms that were significantly enriched in the sets of significantly DE genes. Significant GO terms were defined as having *P*_adj_ < 0.01. Chord plots were constructed using the GOplot package in R.

### Statistical analysis

One-way Student's t-tests were used to determine the significance of the alamarBlue and AV–PI assays. For RNAseq differential expression, the Wald test and a Bonferroni adjustment were used to determine adjusted *P*-values. The linear regression of AV and γH2AX data was evaluated using Pearson coefficient and *P* statistics. One-way ANOVA tests were used to calculate the significance for γH2AX intensities and all morphological comparisons across cell clusters including Shannon Entropy.

## Supplementary Material

pgad415_Supplementary_Data

## Data Availability

The authors declare that all the data supporting the findings of this study are either available within the paper and its Supplementary material or available in public repositories. Raw sequencing data are available at the National Center for Biotechnological Information (NCBI) short read archive (SRA) under BioProject Accession no. PRJNA954385. Scripts for morphological analyses can be found at https://github.com/pkamat22/PM_MORPH.

## Preprint

This manuscript was posted on a preprint: https://doi.org/10.1101/2023.05.17.541204.
