# Supplementary material for: Particulate matter composition drives differential molecular and morphological responses in lung epithelial cells
Source: PNAS Nexus. 2023 Dec 28;3(1):pgad415. doi: 10.1093/pnasnexus/pgad415 (PMC10754159; doi:10.1093/pnasnexus/pgad415)
Supplement: pgad415_Supplementary_Data [file pgad415_supplementary_data.zip › PNASNEXUS-PNASNEXUS-2023-00713R-s18.docx]

|  |  |  |
| --- | --- | --- |
| **Nuclear** | **Cellular** | **Definition** |
| Nuclear Area | Cellular Area | Number of Pixels in a Region |
| Nuclear Bounding Box Area | Cellular Bounding Box Area | Area of a box that surrounds the object |
| Nuclear Compactness | Cellular Compactness | The mean squared distance of the object’s pixels from the centroid divided by the area. A filled circle will have a compactness of 1, with irregular objects or objects with holes having a value greater than 1 |
| Nuclear Eccentricity | Cellular Eccentricity | The eccentricity of the ellipse that has the same second-moments as the region. The eccentricity is the ratio of the distance between the foci of the ellipse and its major axis length. The value is between 0 and 1. (0 and 1 are degenerate cases; an ellipse whose eccentricity is 0 is actually a circle, while an ellipse whose eccentricity is 1 is a line segment.) |
| Nuclear Equivalent Diameter | Cellular Equivalent Diameter | The diameter of a circle or sphere with the same area as the object. |
| Nuclear Euler Number | - | The number of objects in the region minus the number of holes in those objects |
| Nuclear Extent | Cellular Extent | The proportion of the pixels (2D) in the bounding box that are also in the region |
| Nuclear Form Factor | Cellular Form Factor | Calculated as 4*π*Area/Perimeter2. Equals 1 for a perfectly circular object. |
| Nuclear Major Axis Length | Cellular Major Axis Length | The length (in pixels) of the major axis of the ellipse that has the same normalized second central moments as the region |
| Nuclear Maximum Feret Diameter | Cellular Maximum Feret Diameter | The Feret diameter is the distance between two parallel lines tangent on either side of the object (imagine taking a caliper and measuring the object at various angles). The maximum Feret diameter is the largest possible diameter. |
| Nuclear Maximum Radius | Cellular Maximum Radius | The maximum distance of any pixel in the object to the closest pixel outside of the object. |
| Nuclear Mean Radius | Cellular Mean Radius | The mean distance of any pixel in the object to the closest pixel outside of the object |
| Nuclear Median Radius | Cellular Median Radius | The median distance of any pixel in the object to the closest pixel outside of the object. |
| Nuclear Minimum Feret Diameter | Cellular Minimum Feret Diameter | The Feret diameter is the distance between two parallel lines tangent on either side of the object (imagine taking a caliper and measuring the object at various angles). The minimum Feret diameter is the smallest possible diameter. |
| Nuclear Minor Axis Length | Cellular Minor Axis Length | The length (in pixels) of the minor axis of the ellipse that has the same normalized second central moments as the region |
| Nuclear Perimeter | Cellular Perimeter | The total number of pixels around the boundary of each region in the image. |
| Nuclear Solidity | Cellular Solidity | The proportion of the pixels in the convex hull that are also in the object, i.e., *ObjectArea/ConvexHullArea*. |
