# Supplementary material for: Particulate matter composition drives differential molecular and morphological responses in lung epithelial cells
Source: PNAS Nexus. 2023 Dec 28;3(1):pgad415. doi: 10.1093/pnasnexus/pgad415 (PMC10754159; doi:10.1093/pnasnexus/pgad415)
Supplement: pgad415_Supplementary_Data [file pgad415_supplementary_data.zip › PNASNEXUS-PNASNEXUS-2023-00713R-s01.docx]

**
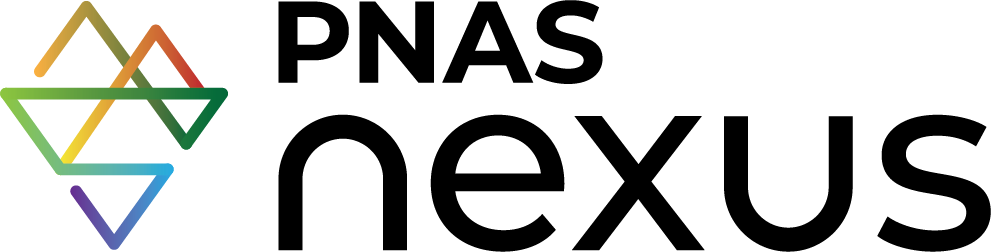
**

**Supplementary Information for**

Particulate matter composition drives differential molecular and morphological responses in lung epithelial cells

Sean M. Engels^1^, Pratik Kamat^2^, G. Stavros Pafilis^1^, Yukang Li^3^, Anshika Agrawal^2^, Daniel J. Haller^4^, Jude M. Phillip^2,5-7*^, Lydia M. Contreras^1,8*^

1. McKetta Department of Chemical Engineering, University of Texas at Austin, Austin, Texas, 78712

2. Department of Chemical and Biomolecular Engineering, Johns Hopkins University, Baltimore, Maryland, 21218

3. Department of Biology, Johns Hopkins University, Baltimore, Maryland 21218

4. Department of Chemical and Biomolecular Engineering, North Carolina State University, Raleigh, North Carolina, 27606

5. Institute for Nanobiotechnology, Johns Hopkins University, Baltimore, Maryland, 21218

6. Department of Biomedical Engineering, Johns Hopkins University, Baltimore, Maryland 21218

7. Department of Oncology, Sidney Kimmel Comprehensive Cancer Center, Baltimore, Maryland, 21231

8. Institute for Cellular and Molecular Biology, The University of Texas at Austin, Austin, Texas, USA

Lydia Contreras and Jude Phillip

Email: [lcontrer@che.utexas.edu](mailto:lcontrer@che.utexas.edu) and [jphillip@jhu.edu](mailto:jphillip@jhu.edu)

**This PDF file includes:**

Figures S1 to S16

Table S1

**Other supplementary materials for this manuscript include the following:**

Datasets S1 to S13

**Fig. S1.** Relative alamarBlue signal following 1.5h or 24h exposures to Diesel Exhaust PM. Culture media without cells was also tested to ensure the Diesel PM was not directly interfering with the assay. Values are percentages of the alamarBlue fluorescence signal relative to unexposed control cells (n=7 replicates, error bars represent one standard deviation). Given that a 1.5h exposure yields significant increases in signal relative to the unexposed control cells, these results show that increased metabolic processing of chemical constituents from the Diesel PM appears to have a major impact on the alamarBlue assay. All cellular Diesel exposure conditions have a significant increase in alamarBlue signal (p<0.001) as measured by a one-way Student’s T-test.

**Fig S2.** High resolution microscopy images of cells from the (**A**) Control, (**B**) Urban 125μg/mL, or (**C**) Urban 500μg/mL exposure conditions captured using a 60x objective. Actin (red), nuclei (blue), and γH2AX (green). Each image is displayed with the same bit range to show the increase in intensity of γH2AX as PM exposure concentration increases.

**Fig. S3.** Flow cytometry analysis of cellular apoptosis. (**A**) Flow cytometry analysis of Annexin V-Propidium Iodide apoptosis assay following PM exposure. A.V+/P.I.- and A.V+/P.I.+ represent early- and late-stage apoptotic cells respectively, A.V-/P.I.+ represents dead cells (n=3, ≥10,000 cells per measurement, error bars represent the standard error of the mean). (**B-H**) Representative flow cytometry scatter plots for analysis of cells following 24h exposure to different PM types and concentrations (500 or 125 µg/mL). displaying the distribution of cells according to Annexin V-FITC intensity (x axis) and propidium iodide intensity (y axis).

**Fig. S4.** Overlap of differentially expressed genes between the tested exposure concentrations. Venn diagrams of genes differentially upregulated (Up) and downregulated (Down) at the 125µg/mL and 500µg/mL concentrations for (**A**) Diesel Exhaust, (**B**) Urban, and (**C**) Fine PM exposures. The majority of up and down regulated genes after the 125µg/mL exposures continue to be up and down regulated after the 500µg/mL exposures respectively.

**Fig. S5.** Enriched GO Biological Process terms resulting from high level (500 µg/mL) exposures. Significantly enriched terms (p_adj_<0.01) that are unique to each individual condition are shown in (**A-C**): (**A**) Urban, (**B**) Diesel, (**C**) Fine. Terms that are shared among two or more conditions and represent a common response are shown in (**D**). Note, no additional terms were significant and unique to the Urban or Diesel conditions. Complete GO analysis can be found in Supplemental Datasets S8-13.

**Fig. S6.** Chord plots displaying the relationships between enriched GO Terms and the associated genes for the 125µg/mL Fine (**A**) and Urban (**B**) exposure conditions. Genes are listed in descending order of differential expression, and the magnitude of gene differential expression is displayed as a color scale of the Log_2_ Fold Change value next to each gene name. A connection between a gene and pathways indicates the gene is involved in that pathway. Genes involved in fewer than 4 pathways are omitted, and pathways with fewer than 3 differentially expressed genes are omitted.

**Fig. S7.** Formation of three cluster groups based on morphological features. (**A**) The 10 k-means clusters that are used to define cell morphology can be further grouped into 3 cluster groups (CG1-3) using Ward based clustering. (**B**) Dendrogram displaying how the clusters were grouped using the 33 measured morphological parameters. The values listed are measured in pixels or unitless ratios.

**Fig. S8.** Distribution of cells in each exposure condition across the UMAP space. UMAP visualization of the 33 measured morphological parameters for each cell in every condition. UMAP-1 (X-axis) was *negatively* correlated with size and UMAP-2 (Y-axis) was *positively* correlated with cell elongation, or linearity. K-means clustering was applied to cluster cells of similar morphologies. The plots on the right show the distribution of cells from each respective exposure condition in red in the UMAP space.

**Fig. S9.** Changes in viable cell counts as a result of PM and cadmium supplementation exposure. Cell counting was performed using the Trypan Blue exclusion method. A hemocytometer was used for counting n=4 replicates, error bars represent one standard deviation.

**Fig. S10.** Cell viability following 24h exposures to different PM types and concentrations supplemented with lead acetate (0-250 µM Pb). Values are percentages of viable cells relative to unexposed control cells as measured with the alamarBlue assay (n=6, error bars represent one standard deviation).

**Fig. S11.** Heatmaps displaying the fractions of cells across the 10 k-means morphological clusters for each PM exposure condition with cadmium supplementation.

**Fig. S12.** Heatmaps displaying the enrichment in number of cells in the three morphology cluster groups for each clonal population. Each heatmap represents morphological distributions from different conditions, either (**A**) baseline morphology, or morphology following the (**B**) 125µg/mL or (**C**) 500µg/mL Urban PM exposure.

**Fig. S13.** Contour maps showing the distributions of morphologies of cells for each clonal population across the UMAP space.

**Fig. S14.** The Shannon Entropies are lowered at baseline for each single cell clone population relative to the parental population. Shannon entropies are shown for (**A**) the control unexposed condition, (**B**) post-exposure to 125µg/ml Urban PM, and (**C**) post-exposure to 500ug/ml Urban PM. Error bars represent standard error of the mean.

**Fig. S15.** Primary factor analysis shows the communality of 32 of the 33 morphological parameters are non-zero. This indicates each parameter, excluding the nuclear area_shape_Euler number, is able to capture some degree of variance. A value closer to 1 indicates the parameter accounts for more variance.

**Fig. S16.** Inertia and silhouette values calculated for various numbers of k-means clusters. The optimal number of clusters, 10, was selected for its local minimum in its inertia/silhouette value and for expressing a reasonable number of clusters.

**Table S1.** List of the morphological parameters that were quantified for each cell in the morphological analysis.

**Dataset S1.** Complete compositional data for the three tested particulate matter mixtures, as reported by the National Institute of Standards and Technology (NIST). A dash represents an unreported value.

**Dataset S2.** Differential gene expression data comparing BEAS-2B cells exposed to Diesel Exhaust PM (SRM 2975) for 24 hours at 125ug/ml to unexposed control cells. baseMean: mean of normalized counts, normalized for sequencing depth. Log2FoldChange: The log base 2 transformation of fold changes. A positive value indicates an increase in expression of a gene upon treatment, relative to the untreated control cells, and vise versa for negative values. lfcSE: Standard Error of the L2FoldChange value. stat: Wald statistic (Log2FoldChange/lfcSE). pvalue: Wald test p-value. padj: Benjamini-Hochberg adjusted p-values.

**Dataset S3.** Differential gene expression data comparing BEAS-2B cells exposed to Diesel Exhaust PM (SRM 2975) for 24 hours at 500ug/ml to unexposed control cells. baseMean: mean of normalized counts, normalized for sequencing depth. Log2FoldChange: The log base 2 transformation of fold changes. A positive value indicates an increase in expression of a gene upon treatment, relative to the untreated control cells, and vise versa for negative values. lfcSE: Standard Error of the L2FoldChange value. stat: Wald statistic (Log2FoldChange/lfcSE). pvalue: Wald test p-value. padj: Benjamini-Hochberg adjusted p-values.

**Dataset S4.** Differential gene expression data comparing BEAS-2B cells exposed to Fine PM (SRM 2786) for 24 hours at 125ug/ml to unexposed control cells. baseMean: mean of normalized counts, normalized for sequencing depth. Log2FoldChange: The log base 2 transformation of fold changes. A positive value indicates an increase in expression of a gene upon treatment, relative to the untreated control cells, and vise versa for negative values. lfcSE: Standard Error of the L2FoldChange value. stat: Wald statistic (Log2FoldChange/lfcSE). pvalue: Wald test p-value. padj: Benjamini-Hochberg adjusted p-values.

**Dataset S5.** Differential gene expression data comparing BEAS-2B cells exposed to Fine PM (SRM 2786) for 24 hours at 500ug/ml to unexposed control cells. baseMean: mean of normalized counts, normalized for sequencing depth. Log2FoldChange: The log base 2 transformation of fold changes. A positive value indicates an increase in expression of a gene upon treatment, relative to the untreated control cells, and vise versa for negative values. lfcSE: Standard Error of the L2FoldChange value. stat: Wald statistic (Log2FoldChange/lfcSE). pvalue: Wald test p-value. padj: Benjamini-Hochberg adjusted p-values.

**Dataset S6.** Differential gene expression data comparing BEAS-2B cells exposed to Urban PM (SRM 1648a) for 24 hours at 125ug/ml to unexposed control cells. baseMean: mean of normalized counts, normalized for sequencing depth. Log2FoldChange: The log base 2 transformation of fold changes. A positive value indicates an increase in expression of a gene upon treatment, relative to the untreated control cells, and vise versa for negative values. lfcSE: Standard Error of the L2FoldChange value. stat: Wald statistic (Log2FoldChange/lfcSE). pvalue: Wald test p-value. padj: Benjamini-Hochberg adjusted p-values.

**Dataset S7.** Differential gene expression data comparing BEAS-2B cells exposed to Urban PM (SRM 1648a) for 24 hours at 500ug/ml to unexposed control cells. baseMean: mean of normalized counts, normalized for sequencing depth. Log2FoldChange: The log base 2 transformation of fold changes. A positive value indicates an increase in expression of a gene upon treatment, relative to the untreated control cells, and vise versa for negative values. lfcSE: Standard Error of the L2FoldChange value. stat: Wald statistic (Log2FoldChange/lfcSE). pvalue: Wald test p-value. padj: Benjamini-Hochberg adjusted p-values.

**Dataset S8.** Gene Ontology Biological Process Terms from the exposure to Diesel Exhaust PM at a concentration of 125ug/ml for 24h.

**Dataset S9.** Gene Ontology Biological Process Terms from the exposure to Diesel Exhaust PM at a concentration of 500ug/ml for 24h.

**Dataset S10.** Gene Ontology Biological Process Terms from the exposure to Fine PM at a concentration of 125ug/ml for 24h.

**Dataset S11.** Gene Ontology Biological Process Terms from the exposure to Fine PM at a concentration of 500ug/ml for 24h.

**Dataset S12.** Gene Ontology Biological Process Terms from the exposure to Urban PM at a concentration of 125ug/ml for 24h.

**Dataset S13.** Gene Ontology Biological Process Terms from the exposure to Urban PM at a concentration of 500ug/ml for 24h.
