# Supplementary figures and images for: Particulate matter composition drives differential molecular and morphological responses in lung epithelial cells
Source: PNAS Nexus. 2023 Dec 28;3(1):pgad415. doi: 10.1093/pnasnexus/pgad415 (PMC10754159; doi:10.1093/pnasnexus/pgad415)

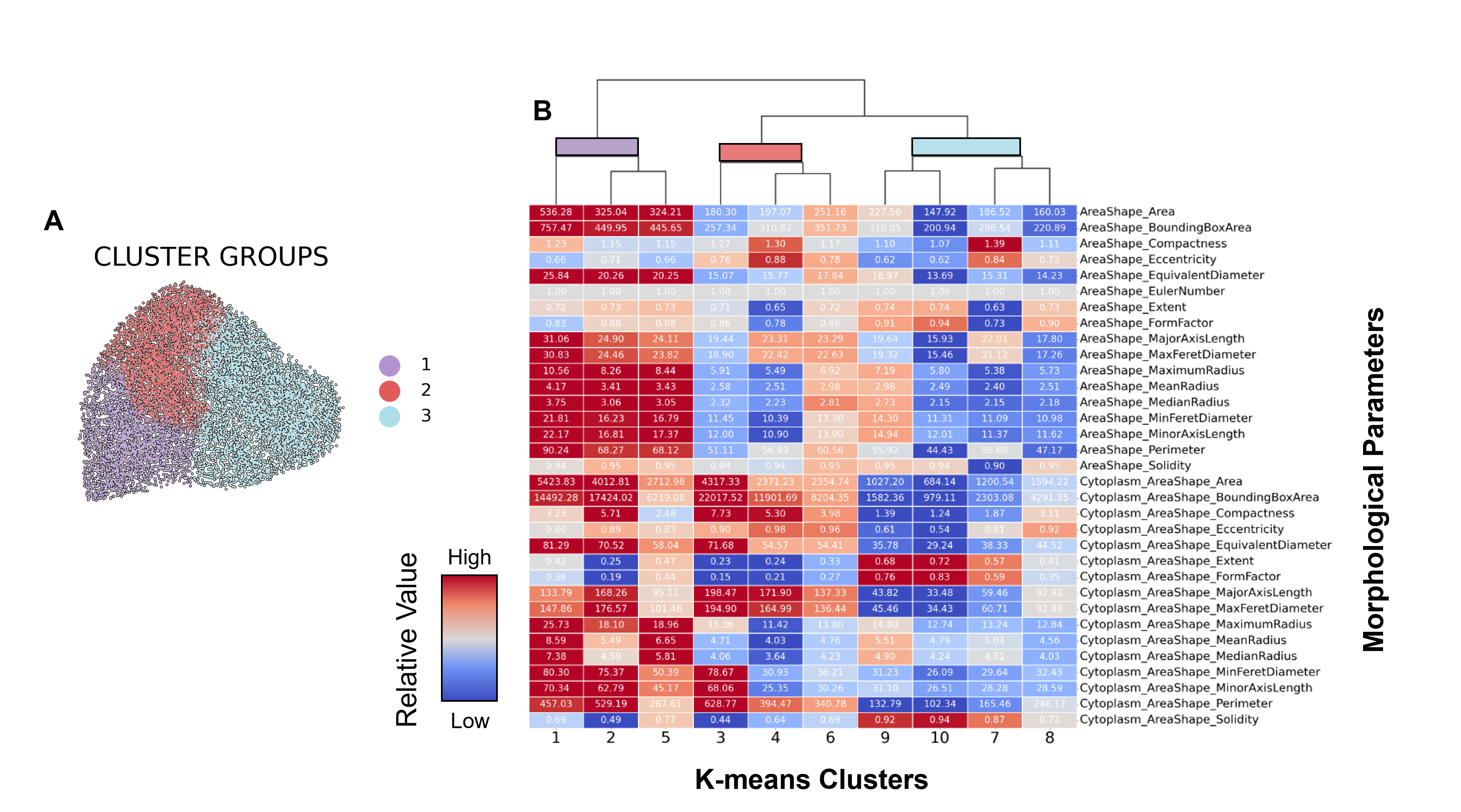

Supplement: pgad415_Supplementary_Data [file pgad415_supplementary_data.zip › PNASNEXUS-PNASNEXUS-2023-00713R-s08.tif]

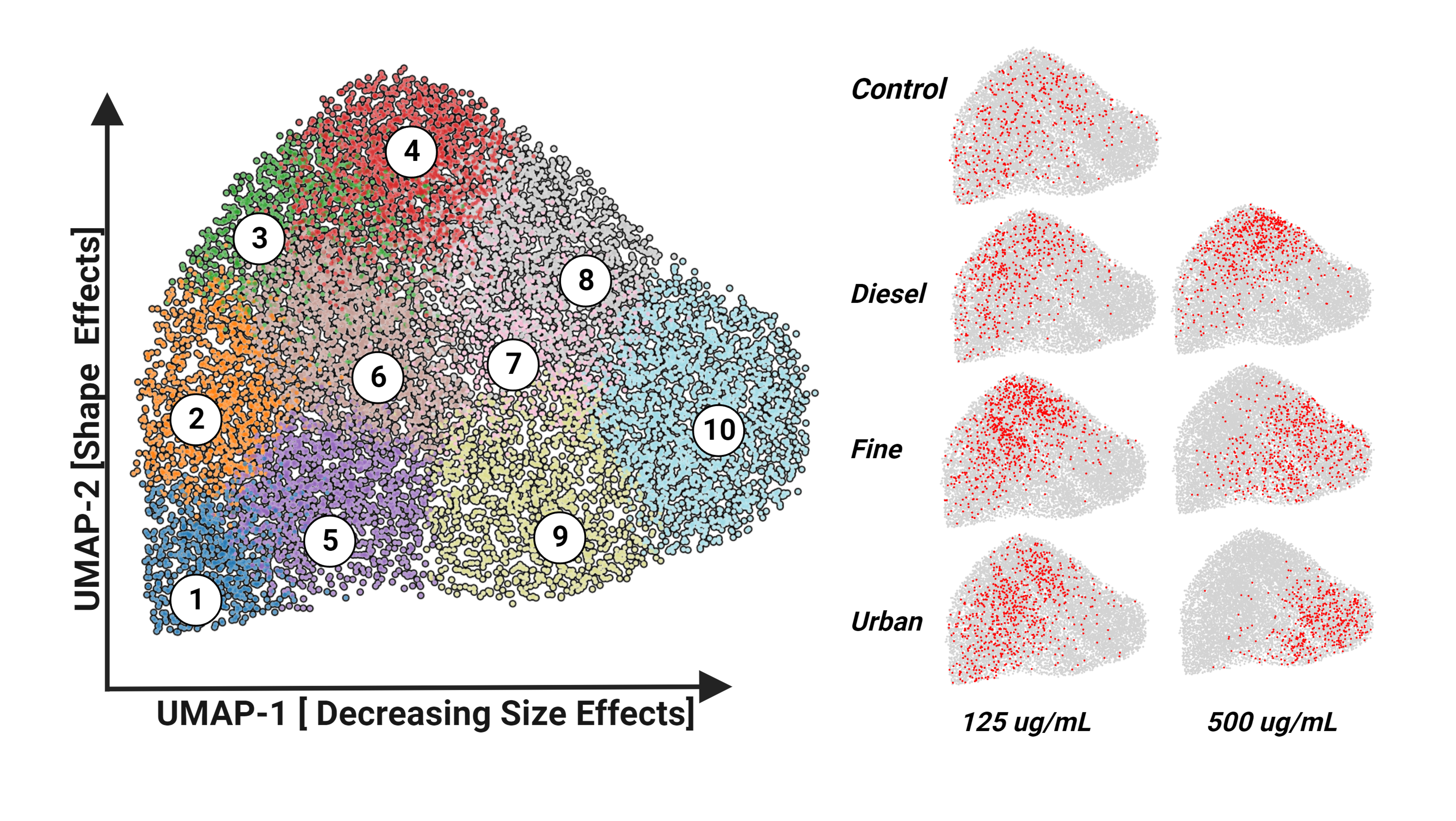

Supplement: pgad415_Supplementary_Data [file pgad415_supplementary_data.zip › PNASNEXUS-PNASNEXUS-2023-00713R-s09.tif]

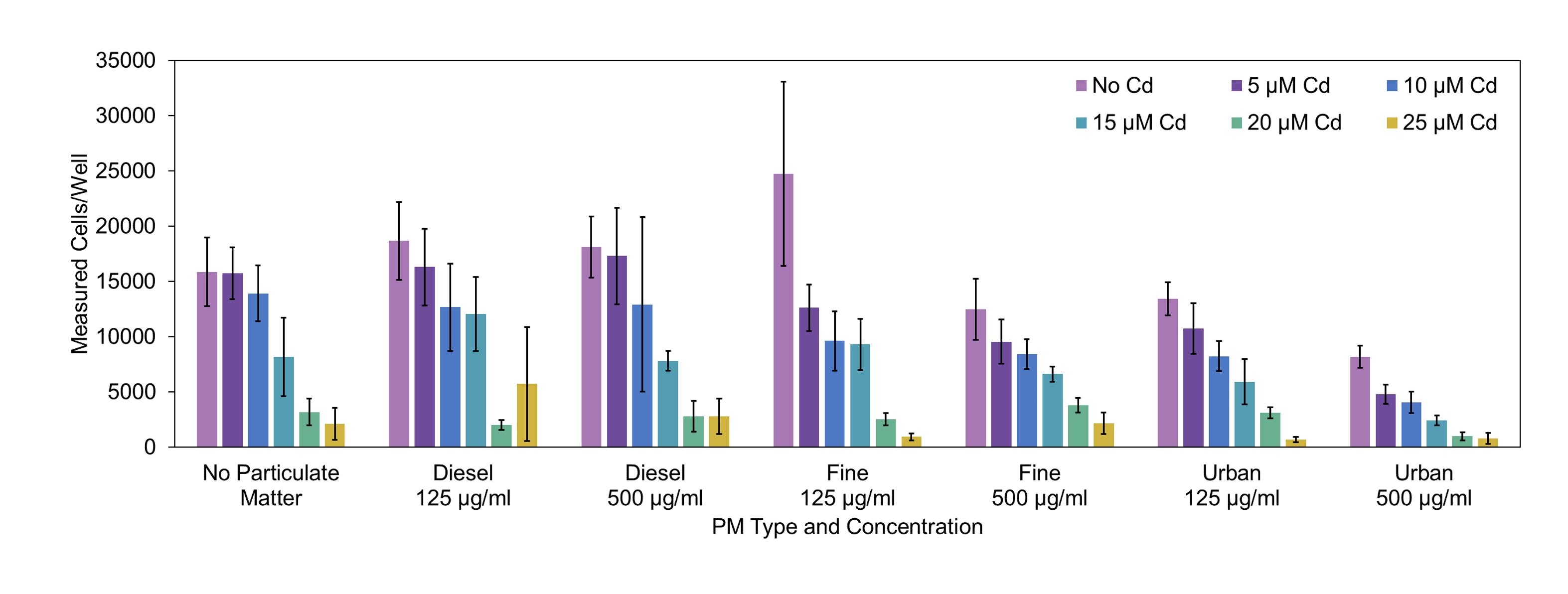

Supplement: pgad415_Supplementary_Data [file pgad415_supplementary_data.zip › PNASNEXUS-PNASNEXUS-2023-00713R-s10.tif]

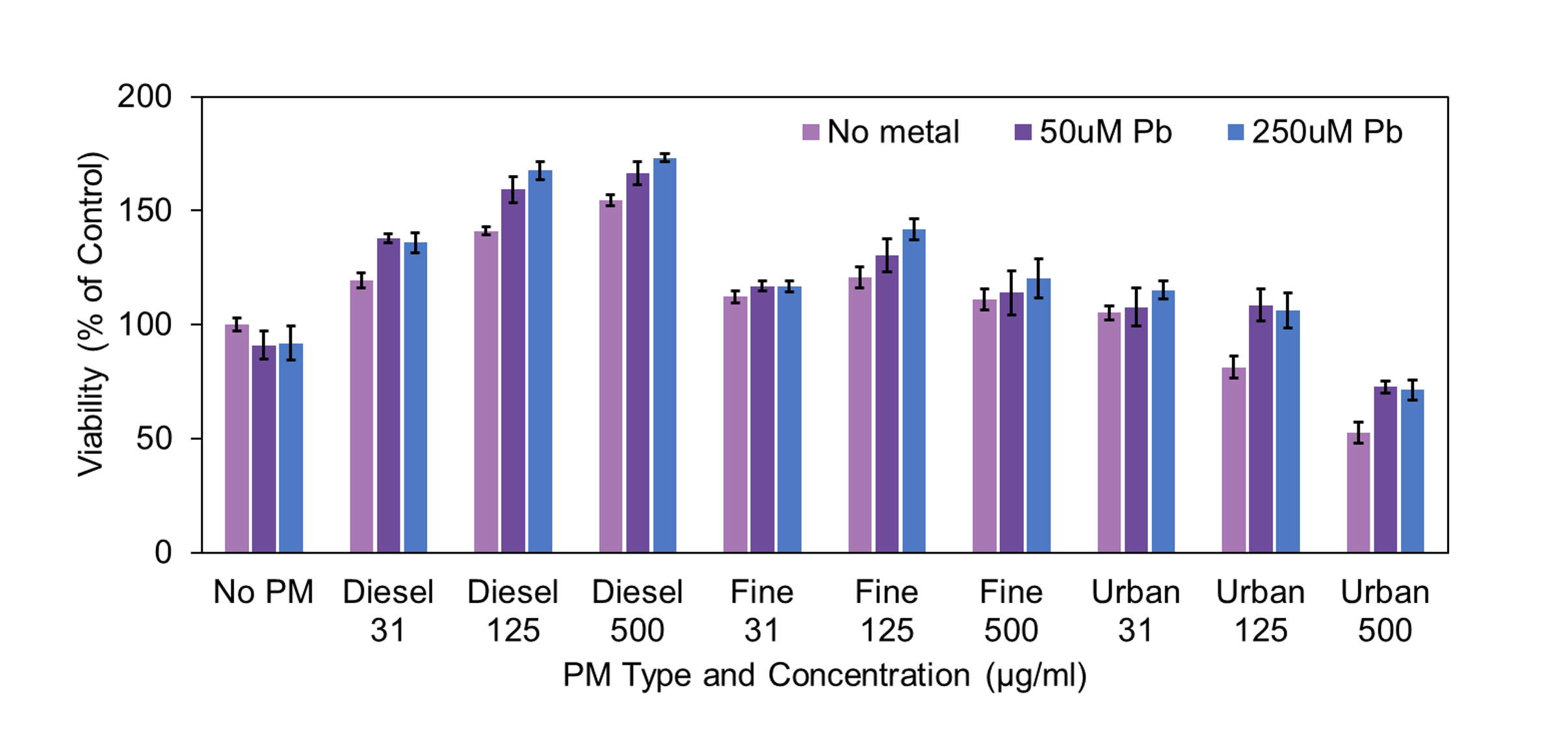

Supplement: pgad415_Supplementary_Data [file pgad415_supplementary_data.zip › PNASNEXUS-PNASNEXUS-2023-00713R-s11.tif]

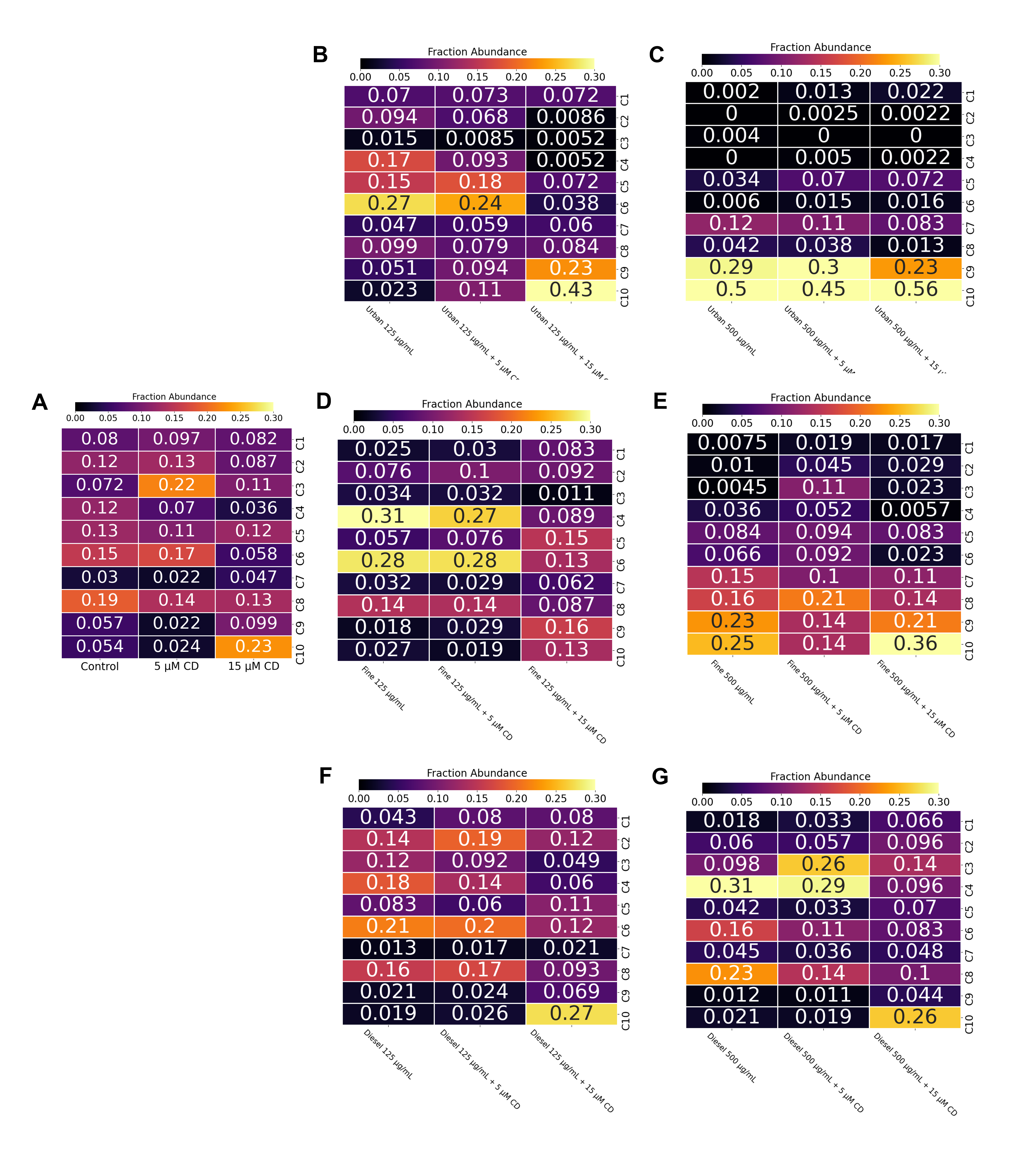

Supplement: pgad415_Supplementary_Data [file pgad415_supplementary_data.zip › PNASNEXUS-PNASNEXUS-2023-00713R-s12.tif]

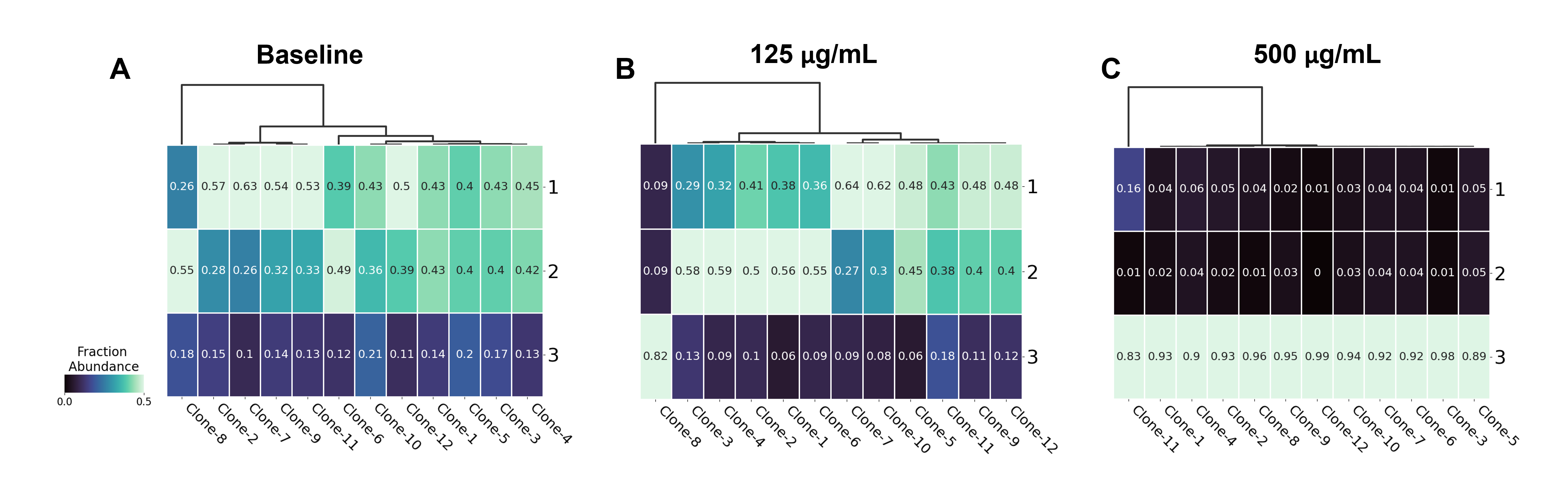

Supplement: pgad415_Supplementary_Data [file pgad415_supplementary_data.zip › PNASNEXUS-PNASNEXUS-2023-00713R-s13.tif]

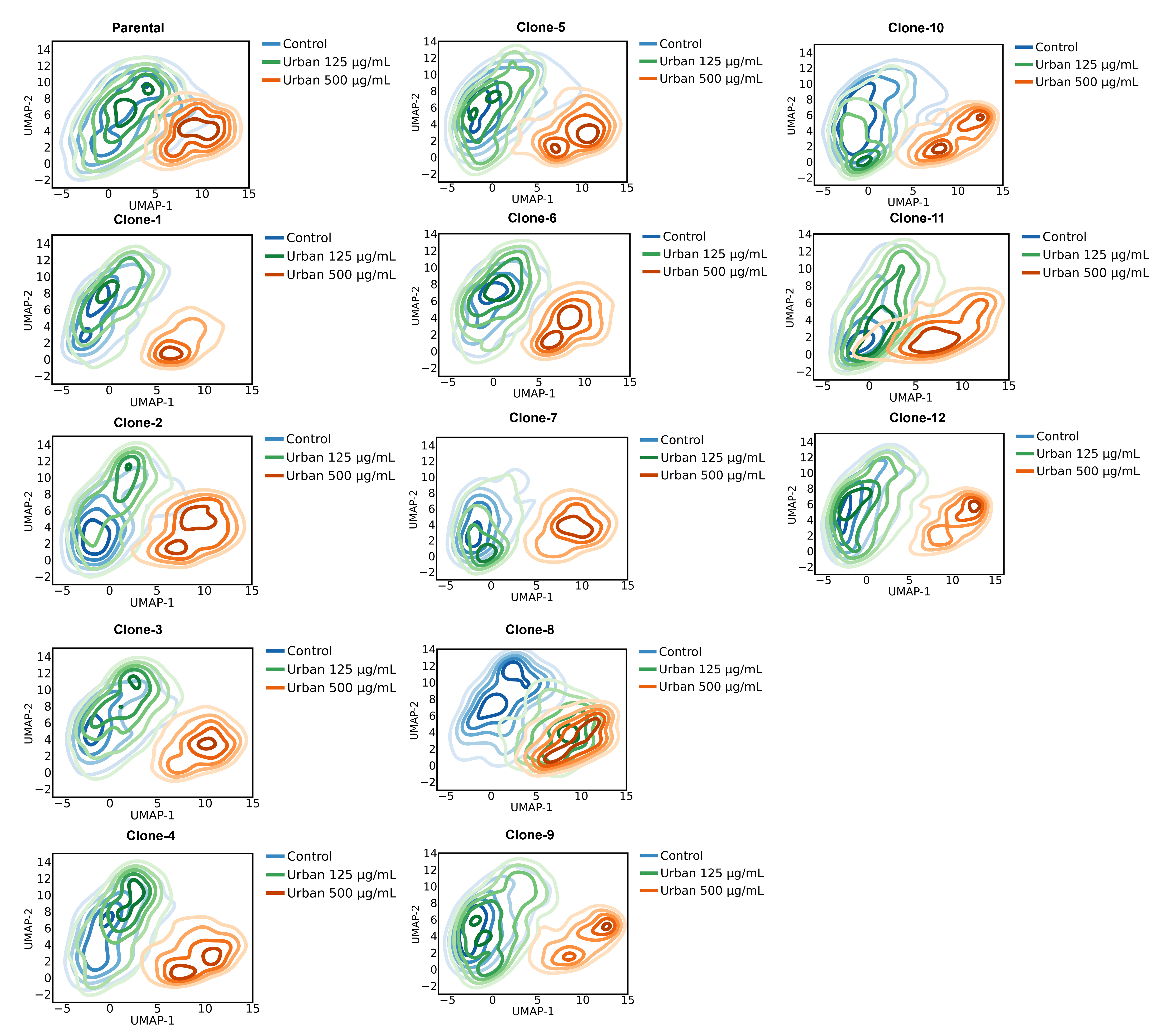

Supplement: pgad415_Supplementary_Data [file pgad415_supplementary_data.zip › PNASNEXUS-PNASNEXUS-2023-00713R-s14.tif]

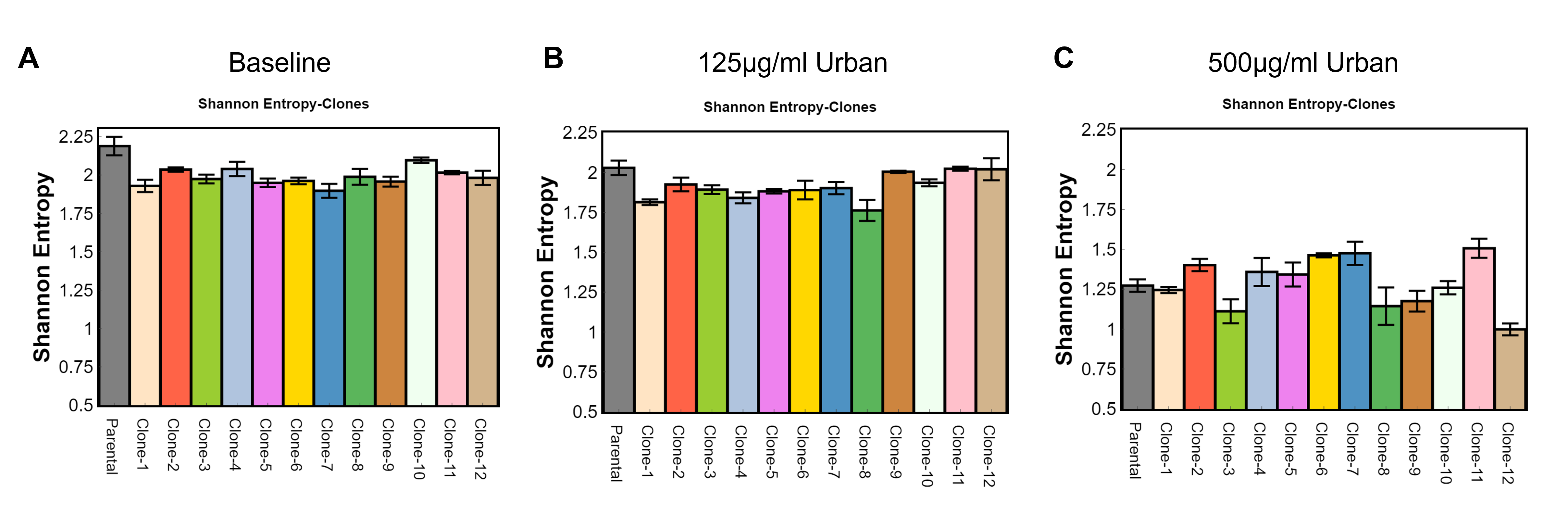

Supplement: pgad415_Supplementary_Data [file pgad415_supplementary_data.zip › PNASNEXUS-PNASNEXUS-2023-00713R-s15.tif]

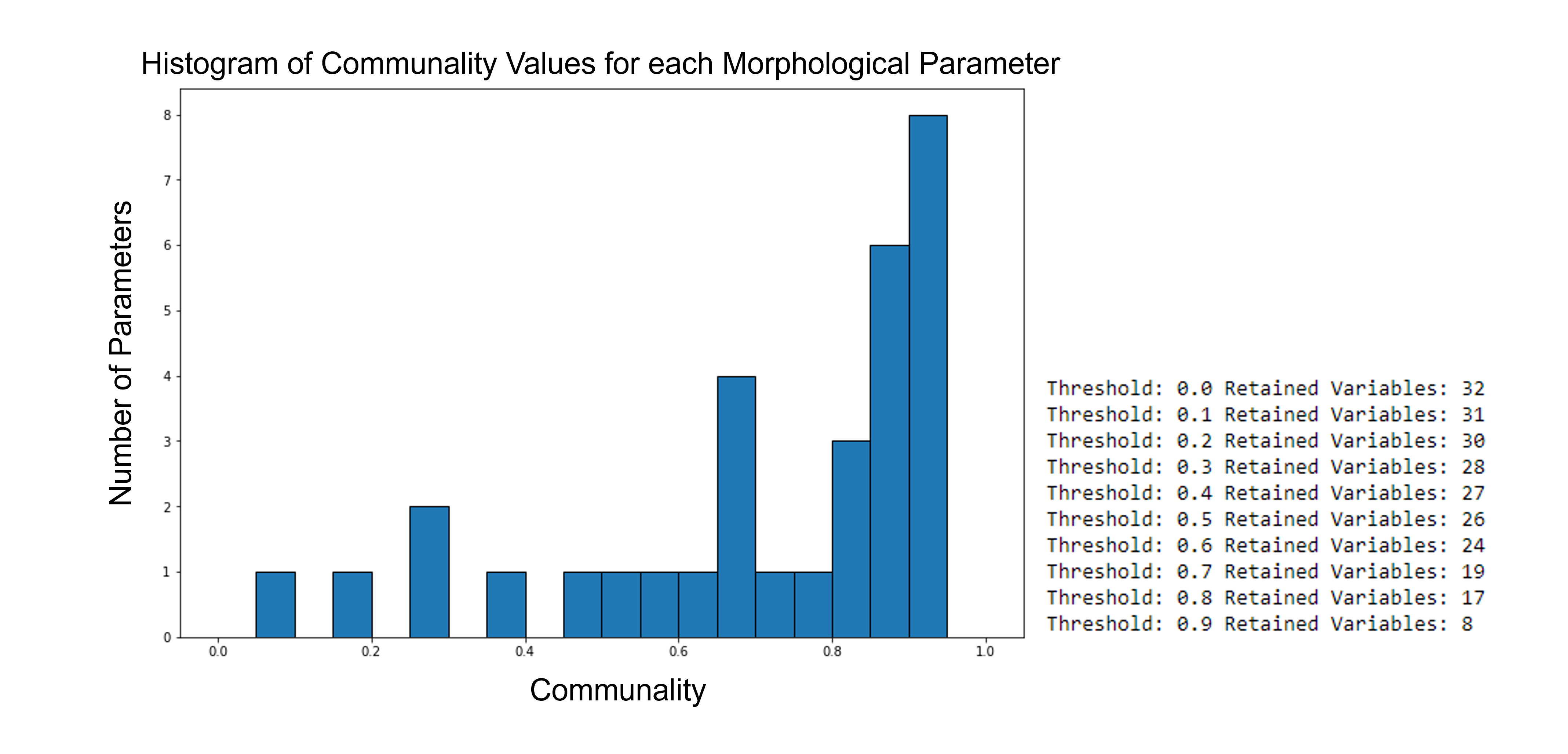

Supplement: pgad415_Supplementary_Data [file pgad415_supplementary_data.zip › PNASNEXUS-PNASNEXUS-2023-00713R-s16.tif]

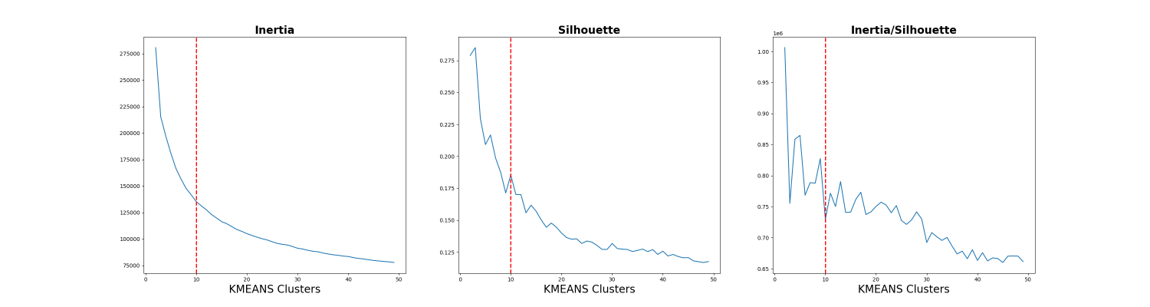

Supplement: pgad415_Supplementary_Data [file pgad415_supplementary_data.zip › PNASNEXUS-PNASNEXUS-2023-00713R-s17.tif]

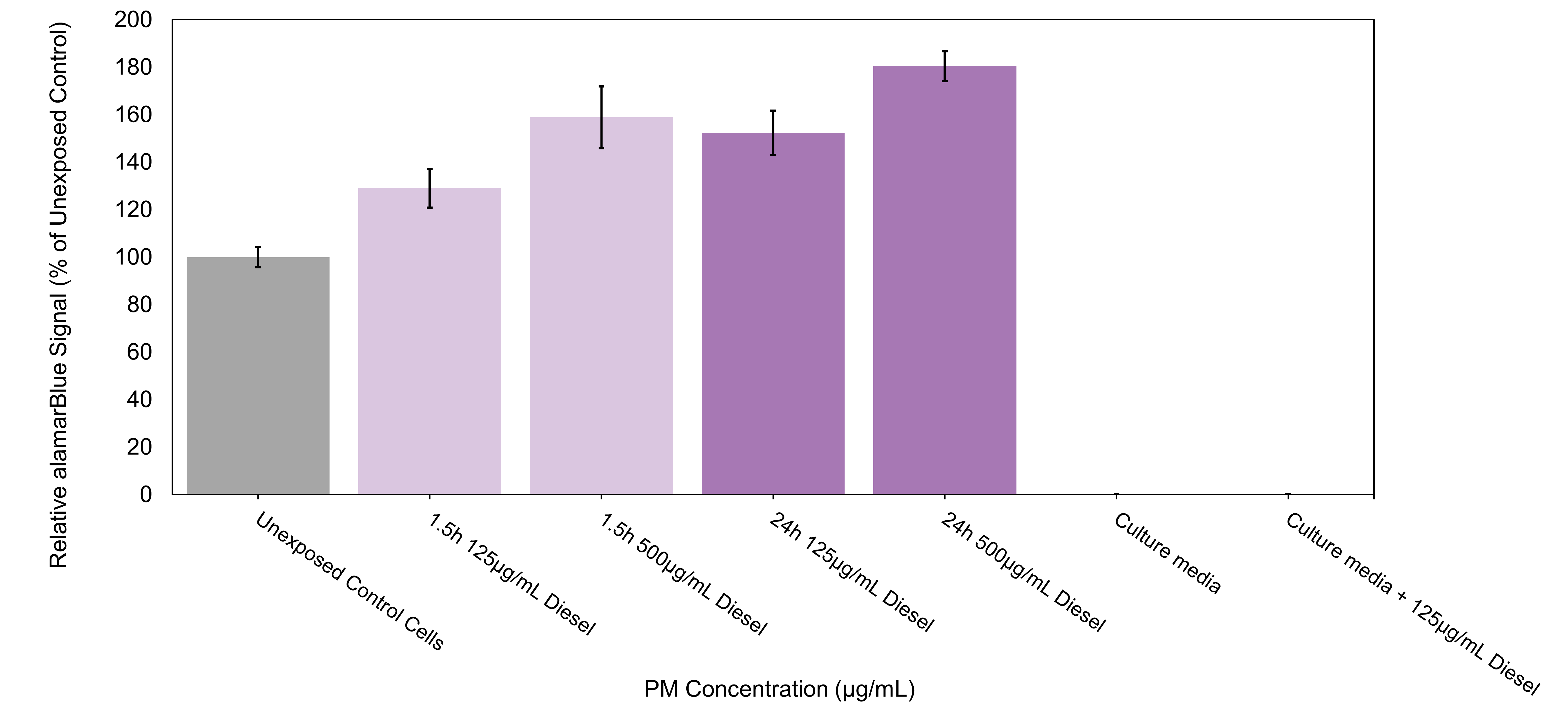

Supplement: pgad415_Supplementary_Data [file pgad415_supplementary_data.zip › PNASNEXUS-PNASNEXUS-2023-00713R-s02.tif]

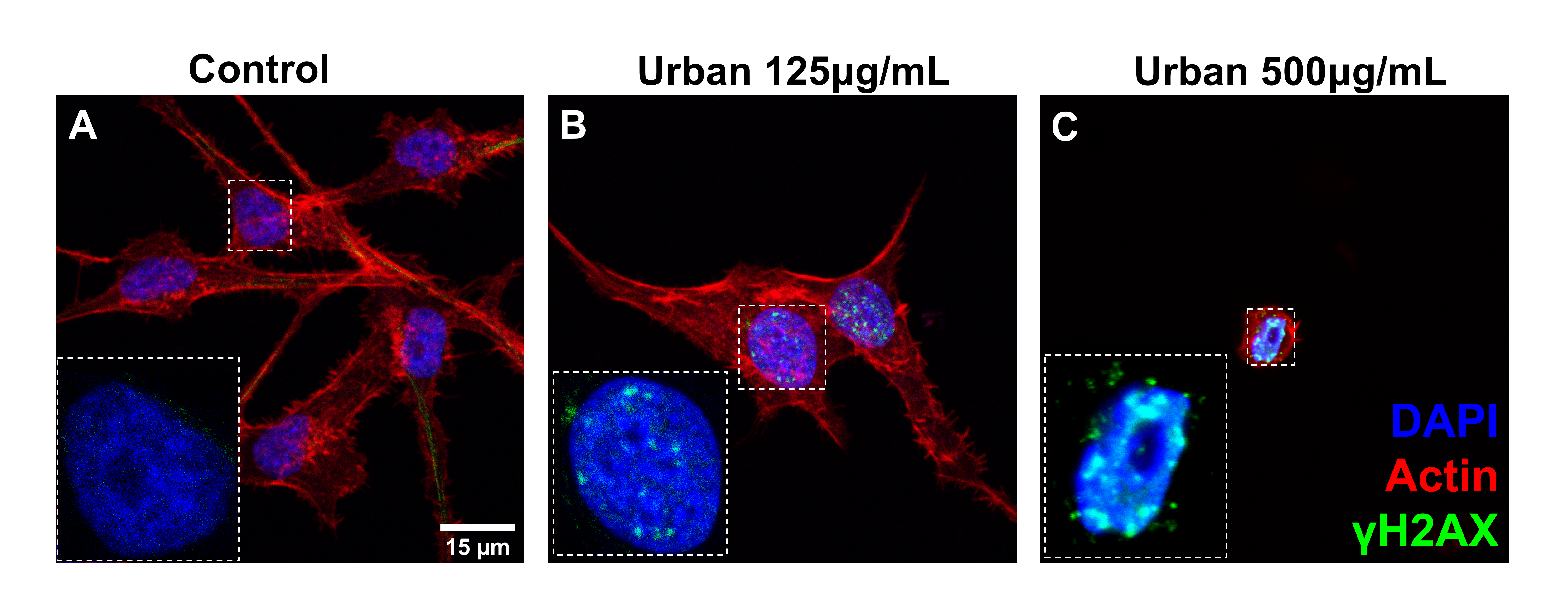

Supplement: pgad415_Supplementary_Data [file pgad415_supplementary_data.zip › PNASNEXUS-PNASNEXUS-2023-00713R-s03.tif]

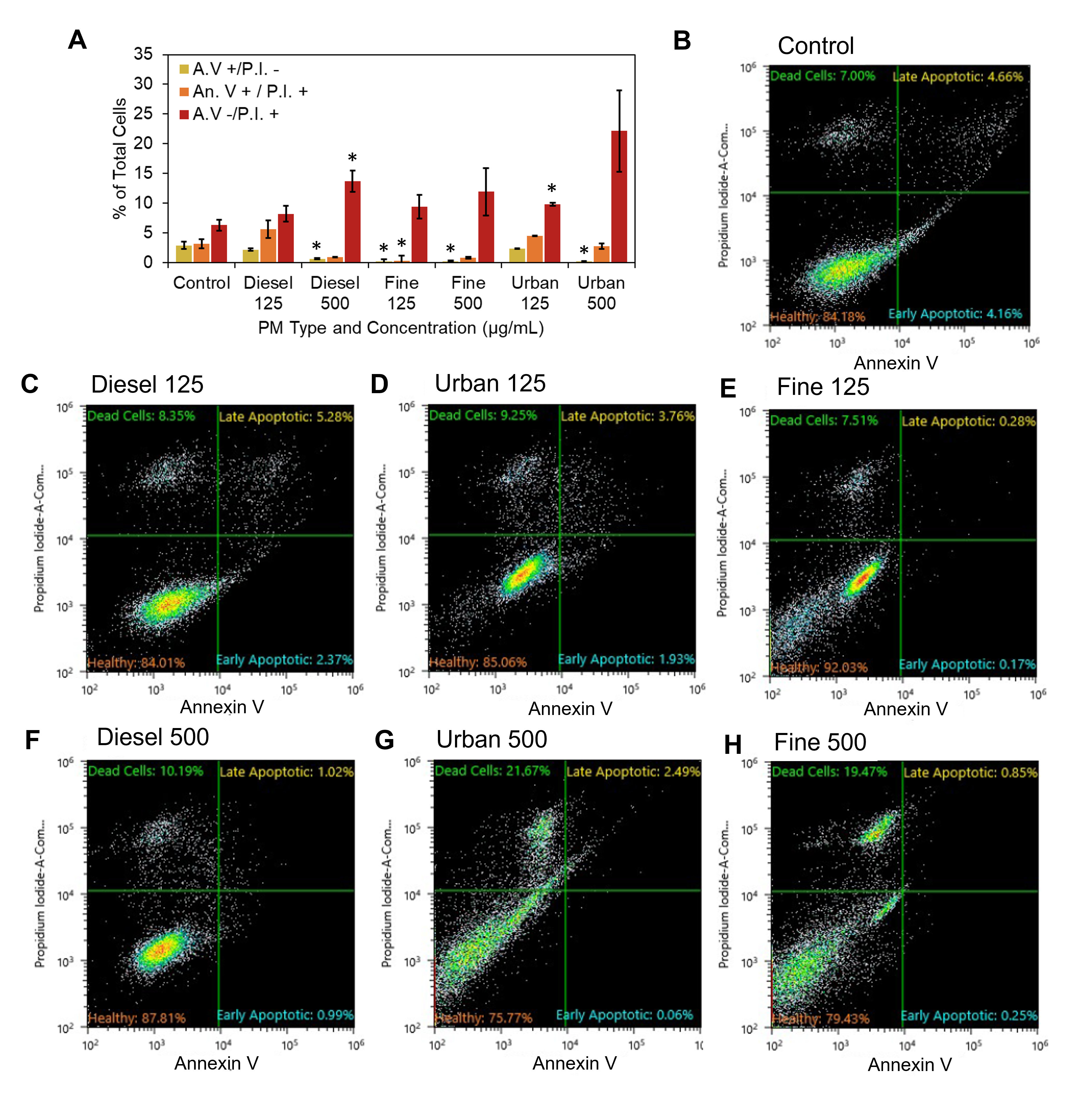

Supplement: pgad415_Supplementary_Data [file pgad415_supplementary_data.zip › PNASNEXUS-PNASNEXUS-2023-00713R-s04.tif]

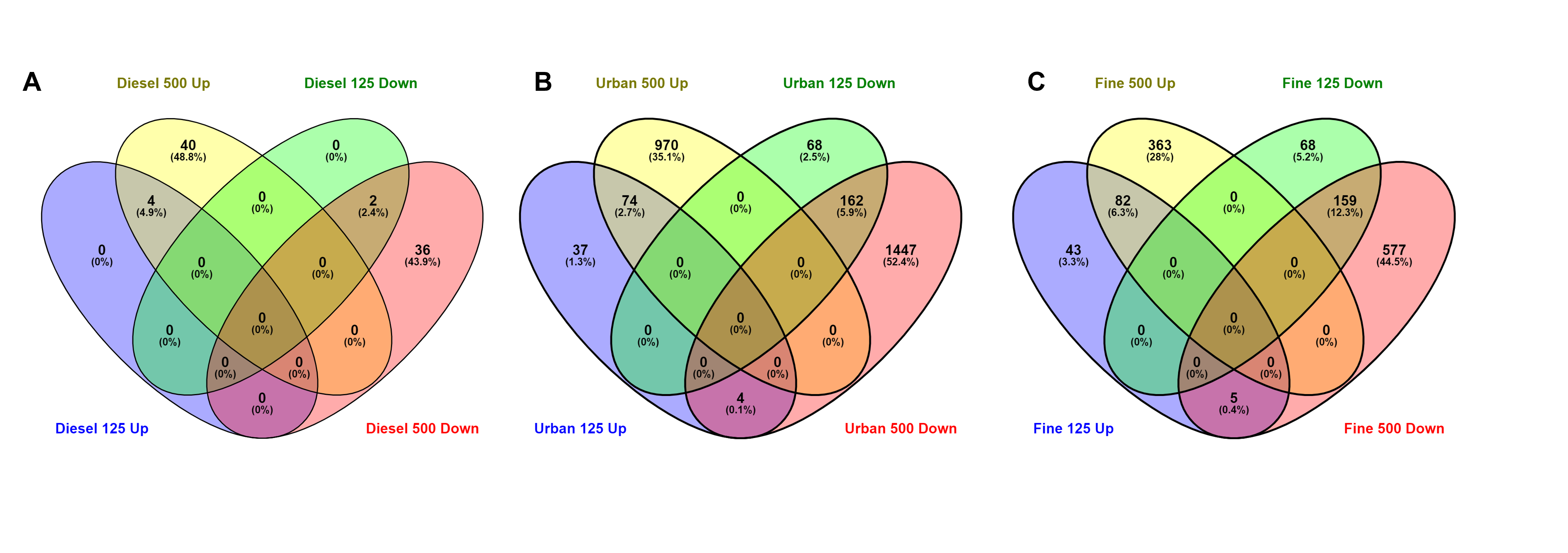

Supplement: pgad415_Supplementary_Data [file pgad415_supplementary_data.zip › PNASNEXUS-PNASNEXUS-2023-00713R-s05.tif]

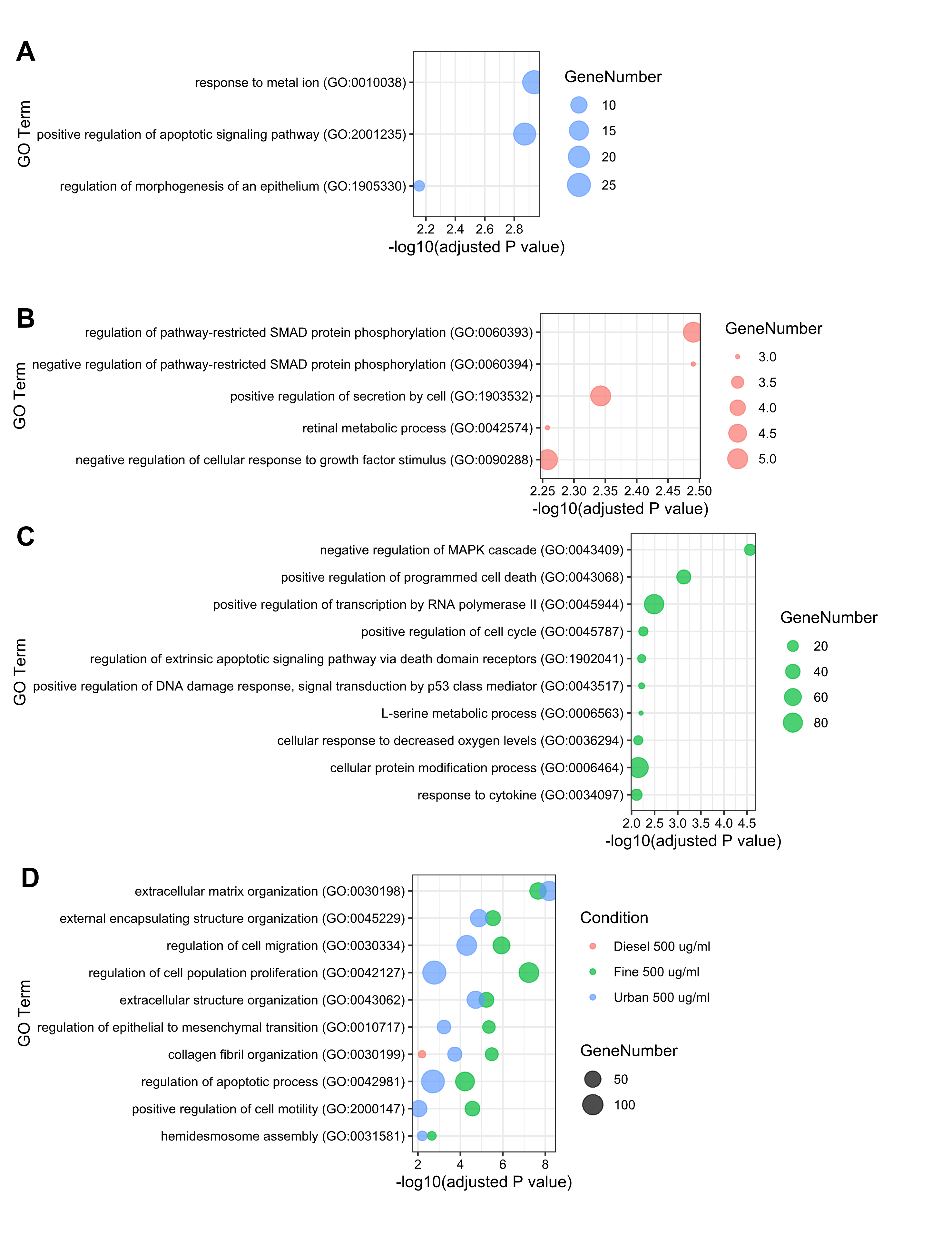

Supplement: pgad415_Supplementary_Data [file pgad415_supplementary_data.zip › PNASNEXUS-PNASNEXUS-2023-00713R-s06.tif]

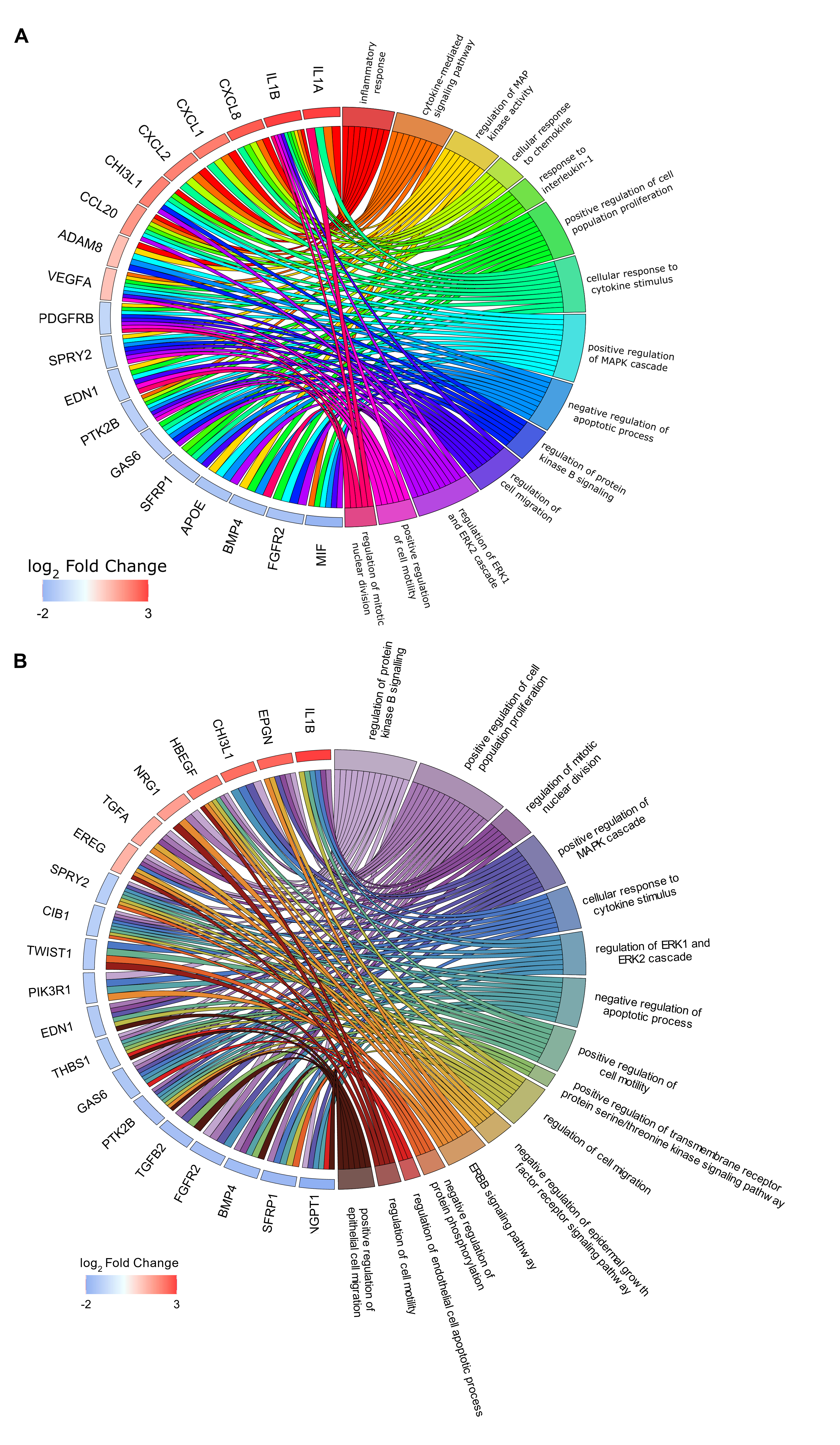

Supplement: pgad415_Supplementary_Data [file pgad415_supplementary_data.zip › PNASNEXUS-PNASNEXUS-2023-00713R-s07.tif]
